# The limitations of bioeconomy LCA studies for understanding the transition to sustainable bioeconomy

**DOI:** 10.1007/s11367-022-02053-w

**Published:** 2022-04-26

**Authors:** Nishtha Talwar, Nicholas M. Holden

**Affiliations:** 1 Agriculture and Food Science Centre, UCD School of Biosystems and Food Engineering, University College Dublin, Belfield, Dublin 4, Ireland; 2 BiOrbic Bioeconomy, SFI Research Centre, Belfield, Dublin 4, Ireland

**Keywords:** Circular bioeconomy, Valorisation, End of life, Bioresource, Biotechnology, Bioecology

## Abstract

**Purpose:**

Transition to bioeconomy requires all actors and stakeholders to measure the impact of systems that use bioresources and technologies to provision society. There are however some challenges with integrating LCA into business development and management, which have important implications for bioeconomy. There have been many LCA studies published in the twenty-first century, but the question must be answered: how useful are these LCA studies to help understand and manage transition to sustainable bioeconomy?

**Method:**

This research used a structured literature review to identify 83 bioeconomy LCA studies published from January 2006 to June 2021 (excluding bioenergy). The studies were analysed for compliance with the ISO 14044 standard, with specific reference to the goal, commissioning perspective, system boundary, function and functional unit, impact methods and categories.

**Results and discussions:**

It was found that more than 85% of the studies reviewed failed to present the required goal statement and a description of the function of the system. Nearly 13% of the studies did not define the system boundary, and only 17% included a full life cycle including raw material extraction, production, use and end-of-life stages. The majority of the LCA studies surveyed from 2006 to 2021 were either (i) not in compliance with the ISO standards or (ii) space and style limitations of the publication process prevented competent practitioners from properly conveying their work. This suggests that the value and integrity of the literature are undermined by not rigorously addressing the first and most important stage of an LCA study.

**Conclusion:**

When interpreting the results, a major shortcoming noted was that most studies did not consider the industrial symbiosis needed between feedstock, technology, primary products, side streams, downstream valorisation and long-term circularity in order to properly understand the transition pathways required. Bioeconomy technologies were imagined as displacers for feedstocks and processes to adapt business as usual, rather than as transformers of the system to a sustainable footing.

**Recommendation:**

If LCA studies are going to provide meaningful information for actors and stakeholders to assess whether a system will be able to operate sustainably, studies should include a full, integrated system, standards should be adhered to and approaches should perhaps go beyond mere eco-efficiency, or doing less harm, as these are not necessarily indicative of sustainability. Historical bioeconomy LCA studies do not provide great insight into the transition to sustainable bioeconomy.

**Supplementary information:**

The online version contains supplementary material available at 10.1007/s11367-022-02053-w.

## Introduction

Bioeconomy, circular economy and circular bioeconomy are emerging as important pathways for social, economic and technical transformation of society to bring it onto a sustainable footing (Karp et al. 2015). The OECD (2009) defined bioeconomy from a techno-economic perspective considering it to be economic activity relating to the invention, development, production and use of biological products and processes. In other words, biological sciences have the potential to add value to products and services (Meyer 2017). In the USA, the Department of Agriculture (USDA) take a broad, dynamic view, defining bioeconomy as “the global industrial transition of sustainably utilizing renewable aquatic and terrestrial renewable resources in energy, intermediate and final products for economic, environmental, social and national security benefits” (The International Advisory Council on Global Bioeconomy 2020). This definition introduces the idea of bioeconomy being a route to sustainability by relying on renewable resources.

In Europe, a resource/environmental view of bioeconomy has emerged (originally focused on resources but more recently also considering the wider environment) encompassing all sectors that rely on biological resources and processing to create value-added products such as food, feed, materials and bioenergy, thus reducing reliance on non-renewable resources while limiting and adapting to climate change, strengthening competitiveness, modernising industry, creating jobs, creating circular economies, minimising waste and supporting healthy ecosystems (European Commission 2018). The EU Bioeconomy Strategy is continuously revised to align with circular economy principles, with the intention that bioeconomy will go beyond the mere replacement for fossil fuels and mineral resources, to maximising reuse and recycling, minimising waste and optimising regeneration to reverse environmental losses and damage and enhance ecosystem functions and biodiversity (Meyer 2017; European Commission 2018).

Regardless of how bioeconomy is defined, key elements are (i) substitution of fossil with biological resources to produce bio-based products and bioenergy, (ii) the idea of using renewable biological resources and (iii) transition to a sustainable economy. The concept of bioeconomy goes beyond biomass flows and is starting to merge with circular economy (Ubando et al. 2020). In the concept of circular bioeconomy and the inverted waste hierarchy, energy recovery is a low priority use of biomass (Stegmann et al. 2020). Bugge et al. (2016) reviewed current definitions of bioeconomy, from which three different stakeholder visions emerged. The *biotechnology vision* focusses on biotechnology research including technologies reliant on microbiology (e.g. fermentation), synthetic biology and gene manipulation with commercialisation for production of materials and chemicals as the end goal (The OECD view). The *bioresource vision* focuses on biological raw materials and the creation of new value chains through upgrading and conversion (where it overlaps with the biotechnology vision) (the original European view). The *bioecology vision* is less concerned with the technologies and resources and more interested in the status and consumption of natural capital and the ecosystem functions (Bugge et al. 2016). The European understanding of bioeconomy has evolved to weigh the three perspectives somewhat equally, which has led to public funds for environmental assessment of new technologies and bioresource value chains being spent alongside the funding of innovation (Collins et al. 2018).

Technical developments to enable bioeconomy come with challenges from a bio-ecological perspective. Foremost is how to remove the reliance on fossil fuel while implementing energy intensive biological processes (Dietz et al. 2018), but the question of sustainable supply of bioresources also looms large. A recent estimate (Transport & Environment and BirdLife International 2016) found that the supply of sustainable biomass in Europe (152 Mtoe) would leave a 15–21% shortfall below EU bioenergy use by 2030, which will represent < 50% of EU energy demand in 2030 (Greenpeace International 2015). This implies that meeting EU energy demand using bioresources would require unsustainable exploitation of biomass or resources beyond the geographical limits of the EU. Add to this, the desire to use bioresources to make materials, even allowing for a very efficient circular economy, even then marrying the visions of bioeconomy will prove very difficult. (McCormick and Kautto 2013). If the transition to bioeconomy will help achieve the Sustainable Development Goals (SDGs), attention will have to be paid to both the production side and the consumption side of the bioeconomy equation (SDG12), while also contributing to zero hunger (SDG2), clean water (SDG6), energy (SDG7), a decent living (SDG8), industrial resilience (SDG9), climate action (SDG13) and life on earth (SDG14 and SDG15) (Hakovirta et al. 2020). To design effective enabling and regulatory governance frameworks for bio-based transformation, policy makers need to identify potential technologies and their associated environmental gains and losses (Chandrakumar and McLaren 2018). Those technologies being commercialised for the bioeconomy need to be implemented in a system (environment bioresource technology environment) that is sustainable, which means quantitative assessment tools are required, because *if we cannot measure, we cannot manage*. One tool that is currently used to provide such “measures” is life cycle assessment (LCA).

Life cycle assessment is a framework that evaluates the environmental impacts of a product or service from cradle to grave (ISO 14044 2006). It has been used to identify a range of potential environmental impacts of a product, process or service from resource extraction (cradle) to production, use, reuse, recycling and final disposal (grave) (Matthews et al. 2014). LCA provides a measure of eco-efficiency (impact per functional unit) rather than the absolute impact of the system function (Bjørn and Hauschild 2013). While technical developments have been proposed to consider absolute impact using concepts like carrying capacity (Bjørn et al. 2016), which reflect the bioresource lens of bioeconomy, the vast majority of LCA studies have focused on eco-efficiency. A benefit of using LCA is the necessity for “system thinking” resulting in a holistic assessment of the product, process or service (Matthews et al. 2014). This helps stakeholder decision-making around hotspots, energy, costs and types of impacts, all of which should encourage sustainable business planning (ISO 14044 2006). There are however some challenges with integrating LCA into business development and management, which have important implications for the bioeconomy.

1. LCA studies tend to be retrospective (Sandén and Karlström 2007). This means they rely on historical data, and so an innovation has to be made real (money spent, time committed, infrastructure constructed, product sold) for the data to be available for modelling. The concept of prospective LCA has been proposed (Thonemann and Schulte 2019) to overcome this issue, which is particularly important from a biotechnology perspective.
2. Selecting the right type of model. Process-based LCA can be classified into two main approaches, attributional and consequential (Myllyviita et al. 2019). An attributional LCA accounts for a product’s environmental impact and can also be referred to as “descriptive” (McManus and Taylor 2015) or “retrospective” (Guin 2001). The consequential LCA focusses on the consequences of changing a technology and its performance (EC-JRC 2010). Here, the main difference between the two approaches is the nature of the input data used for the analysis (Rønning and Brekke 2013). Both the approaches can help the commissioner in making an informed decision (Weidema et al. 2009). Industry-led standardisation of LCA (e.g. PEF (Bach et al. 2018) and EPD (Durão et al. 2020)) has tended to favour attributional LCA because there is less perceived uncertainty (Rehl et al. 2012) associated with the consequence of a decision (Ronning and Brekke 2014), but this point of view is not universally accepted (Weidema et al. 2009).
3. Dealing with the different perceptions of technology and feedstock combinations, particularly in the bioeconomy. Oldfield et al. (2018) examined two contrasting commissioning stakeholder perspectives of what are regarded as bioeconomy technologies and concluded that very different results would be obtained because stakeholder perspective will result in different decisions about the goal and scope of the study.
4. How to model a system viewed through the biotechnology or bioresource lens, i.e. where the stakeholder’s primary interest is the technology or feedstock rather than the end product of the system (closely related to the third challenge) (Meyer 2017). In the context of bioeconomy, the materials, while important, are perhaps less significant than the products they are used to make. This tends to be a knowledge gap if the system is not viewed holistically. The end product, the market it competes in and how it might be circulated rather than disposed of are critical questions (related to the second challenge and consequential modelling). While LCA is widely used for assessment of bioeconomy for stakeholders looking through both the biotechnology and bioresource lens, if these challenges are bypassed rather than addressed head-on, it may well not offer valuable information for informed management or policy decision-making.

There are many points in the LCA process where decisions will influence the results obtained, whether the results are comparable among studies and how meaningful any recommendations might be (Finnegan et al. 2017). Important considerations include the goal (influencing perspective and application, thus interpretation), aspects of the scope (e.g. type of model, system boundary, circularity, function, functional unit, allocation, impact categories, impact methods) and scale up of inventory data (e.g. from pilot plant to full-scale implementation). A clearly articulated goal statement is a mandatory component of an ISO standard LCA (ISO 14044 2006) and is required by ILCD (ILCD 2010) and most environmental product declarations (Durão et al. 2020). Different actors and stakeholders can perceive the function of a system quite differently, particularly when an unwanted resource is involved, where it might be seen as a waste, requiring an end-of-pipe disposal function, or as a valuable substance, required as a feedstock for a process to make something new (Oldfield et al. 2018). In the first case, the stakeholder perceives the resource flow as having no value, while in the second, the stakeholder perceives it as a valuable substance, essential for the business. This gives rise to different modelling choices that make comparative assessments of bioeconomy studies difficult because choices made by the LCA practitioner can greatly affect the results of the study (as demonstrated by Yan et al. 2011). In view of the need for innovation in bio-based industries on the one hand, and the limitations and potential of LCA on the other, a clear understanding of how LCA influences decision-making is needed. This study focused on work related to the production of high value materials, which has not been considered before in this way. There have been bioenergy LCA reviews (e.g. Muench and Guenther 2013; Ubando et al. 2020); so this aspect of bioeconomy is not considered here. The objective of this research was to understand how LCA studies have been designed and conducted and whether they provide useful information about the sustainability of production systems built on bioeconomy technologies and feedstocks for the supply of chemicals and biomaterials. The approach taken was to review literature and synthesise the methodological choices made to answer the question: Is LCA being used in the best possible manner in the context of the emerging bioeconomy?

## Methodology

**Fig. 1:**
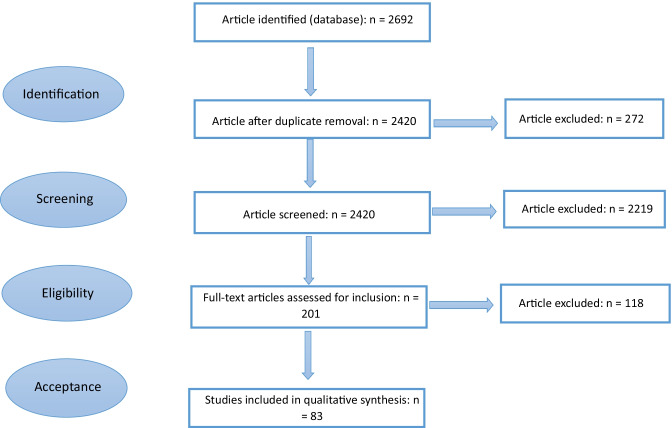
The review process in compliance to Preferred Reporting Items for Systematic Review and Meta-Analysis (PRISMA) guidelines. Source: adapted from Gottinger et al. (2020)

The review of literature was based on the STARR LCA framework (Zumsteg et al. 2012) to ensure consistency in the review. Figure 1 is a flow diagram adopted from Gottinger et al. (2020) and modified as per STARR LCA guidelines to aid understanding of the reviewing process and thus increase the transparency of the study.

For the selection of relevant publications, three databases were used, Scopus, Science Direct and Web of Science. To reduce the risk of selection bias, the search string was set prior to the literature search. The search string used the following words and logical operators: (“life cycle analysis” OR “life cycle assessment” OR “LCA”) AND ((bioeconomy) OR (bio-economy) OR (bio-based bio-economy) OR (bio-based bioeconomy) OR (bio-based product) OR (bioeconomy technology)). This approach identified a large number of publications from the research field: 2420 documents after eliminating duplicates. Title and abstract screening were completed using Rayyan QCRI (https://rayyan.qcri.org/) to exclude review articles, books, book chapters, conference proceedings and studies focused on bioenergy, which left 201 documents. The time frame considered was January 2006 to June 2021.

To select only relevant literature in the final sample, the publication had to meet the following criteria: (i) the study should consist of an environmental life cycle assessment; (ii) it should be focused on bio-based feedstocks, organic wastes and bioprocess conversion technologies to replace fossil products with bio-based products only; and (iii) the research objective of the study should be aimed at the transition towards bioeconomy. With these set criteria, the 201 documents were read in detail, and 83 LCA studies related to bioeconomy technologies and bio-based resources were identified to analyse methodological choices and whether there were implications for the evidence used to manage transition to a sustainable system. The 83 studies were focused on bio-based industries that create innovative, non-food, high value-added products. These ranged from high-value fine chemicals such as pharmaceuticals, cosmetics and food additives to high volume materials such as biopolymers and chemical feedstocks. Food; traditional bio-based products, such as pulp, paper and wood products; and biomass as an energy source were excluded from the study. This limit was set because the intention was to assess the role of LCA in the transition to bioeconomy focusing on maintaining the value of resources, materials and products that could remain in the economy for as long as possible (Leoussis and Brzezicka 2017).

The analysis of the 83 documents focused on the following: (i) methodological choices (Table 1) driven by the goal (reason, application), (ii) the category of feedstock (Table 2), (iii) how the LCA was conducted in terms of commissioning perspective and (iv) the bioeconomy lens through which it was viewed. The underlying research questions for the review were (i) to analyse the consistency of methodological choices and whether they were in line with the ISO 14044 guidelines and (ii) whether methodological choices limited the value of the study in providing information about transitioning to a sustainable bioeconomy. A spreadsheet (available as supplementary material) was compiled to record source references and data for each issue noted in Table 1, and the results were compiled to provide quantitative evidence of how LCA has been applied in the context of bioeconomy. A list of all reviewed publications is included in Annex A.

**Table 1:** Data collection form

Data collection form	
Ref. ID	Natural numbers
Geography	Region specific (Asia, Europe, North America, Australia, Africa)
LCA approach	1) Process based—a) attributional, b) consequential, 2) input output; 3) hybrid
Feedstock	See Table 2
Commissioning perspective	1) Explicitly mentioned; 2) implicated; 3) business as usual; 4) not mentioned
Goal	1) Full goal statement with all the components as per ISO or ILCD guidelines; 2) incomplete goal statement but wrt to the ISO or ILCD guidelines; 3) type of aim or reason for the study is mentioned (addressing a research question); 4) the goal statement has no discernible purpose; 5) goal not defined
Application of goal	This parameter is divided into 5 types: 1) hotspot identification; 2) improvement of the process (environmental, economic, etc.); 3) market (new study, lab scale to large scale); 4) policy related (some explicit reason or already inferred); 5) not defined
Function and functional unit	1) Function and functional unit is defined and complement each other; 2) FU is the function of the study; 3) only functional unit is defined in the study; 4) more than 1 FU; 5) no FU defined
System boundary	5 types: 1) cradle to grave; 2) cradle to gate; 3) gate to gate; 4) cradle to cradle; 5) gate to grave; 6) not defined
Impact categories and methods	1) Multiple impacts with defined methods; 2) single impact with defined methods; 3) multiple impacts without defined methods; 4) single impacts without defined method; 5) no information of impacts and methods provided in the study
Number of impact categories	1) More than 10; 2) at least 5; 3) 3 impacts or more; 4) 2 or 1 impact mentioned; 5) no description
Interpretations^1^	1) Sensitivity analysis; 2) uncertainty analysis; 3) assessment of data quality; 4) recommendations

^1^The characteristics of interpretations were not analysed in detail

**Table 2:** Types of feedstocks

Feedstock	Description
Crop residues and perennial plants	Agricultural residues from dedicated crop production with no value-added use or treated as waste, non-edible biomass such as perennial grasses or lignocellulosic crops, e.g. switch grass or corn stover
Genetically engineered crops	Genetically engineered or systematically bred plant varieties to extract or produce high value-added bio-based products
Marine biomass	Biomass obtained from cultivated macro- or microalgae
Waste or recycled feedstock	Processes using waste or recycled material in closed-loop approaches
Commercialised chemical commodity	Fertilisers, manures, commercial chemicals or chemical materials

## Results

The data compiled from the literature review are summarised in the supplementary materials. The data were analysed using the classification presented in Table 1.

### Geography

The majority of the work was conducted in Europe (63/83). A concentration was observed in Italy (14/83) and the UK (10/83). This could be due to the HORIZON 2020 projects and targets set by the European Union (Bio-based Industries 2014). A few LCA studies were conducted in the USA (7/83) with very small numbers from nine other countries (13/83).

### LCA approach

There are five different types of life cycle approaches: (a) process based, (b) hybrid, (c) social, (e) life cycle costing (LCC) and (f) single issue footprints. Social LCA, LCC and single issue footprints were not included in the scope of this systematic review. Most of the LCA studies were process-based, and within that class, there were further sub-classes of the approach, i.e. attributional, consequential and streamlined. There was 1 hybrid LCA and 11 studies that did not describe the approach sufficiently to classify. Attributional LCA was used by 44/83 studies, while 23/83 studies used consequential LCA and 3 streamlined LCA studies. Off the 23 consequential studies, 5 are prospective with a focus on the future of the technology or process involved. On the other hand, 15/44 attributional LCA studies were retrospective, and only 2/44 were focussed on prospective bioeconomy technologies. Whether a study was retrospective or prospective was inferred from the goal and scope statement and interpretation. Only 1/83 studies used both attributional and consequential LCA approach.

### Feedstocks

**Fig. 2:**
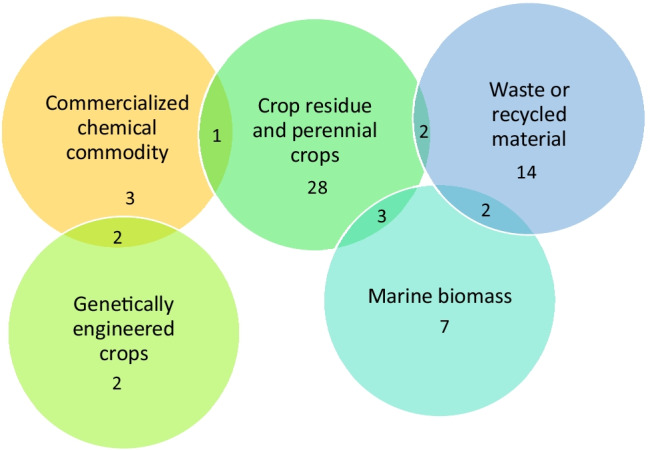
Feedstock and its distribution in the review (numbers represent the number of studies in each category)

The types and overlaps of feedstocks are summarised in Fig. 2. Over half of the studies (38/83) considered primary terrestrial crop feedstocks, of which 10 considered crop residues, 24 considered perennial crops and 4 considered crops that were genetically modified to enhance biomass. There were 18/83 studies that considered a “waste” stream or recovered material, of which 12 were of terrestrial origin. Only 12/83 of the studies considered marine biomass such as cultivated micro- or macroalgae as the feedstock, despite the growing interest in ocean resources. A small number (6/83) of the studies considered chemical materials as the feedstock for a biological technology process. While these cases fall within a very general definition of bioeconomy, they might be excluded using a bio-resource and bio-ecological view point. There were 10/83 studies where more than one feedstock was used, for example to evaluate which feedstock would require least energy for polymer production (Zhang et al. 2018b).

### Commissioning perspective

The commissioner is the entity or person who instigates and usually funds the study. The commissioning perspective is important because it determines the goal (including reason, application and audience). Just over a third of the studies (32/83) did not mention the role of a commissioner, and it was not possible to infer the commissioning perspective. Less than a third of the studies (27/83) clearly identified the commissioner, while it was possible to infer the commissioning perspective, even if it was not clearly stated in just over a third of the studies (24/83).

**Fig. 3:**
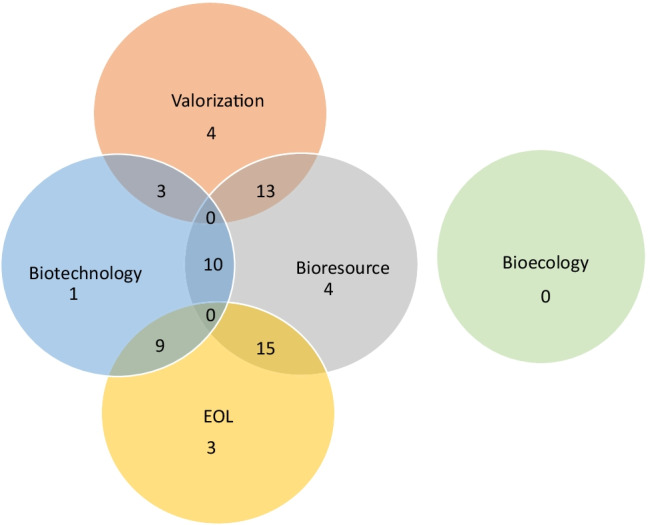
Overlap between stakeholders’ perspective and bioeconomy lens (numbers represent the number of studies in each category)

The stakeholder perspective was also assessed in terms of the bioeconomy lens, i.e. biotechnology, bioresource or bioecology (Fig. 3). The bioeconomy was most commonly viewed through a bioresource lens (42/83) with 13 studies considering valorisation. The goal statement in most of these studies (10/13) considered process improvement as the intended application, and transition from fossil fuel economy to bioeconomy was given an overall emphasis. An end-of-life perspective was also common (15/42), with process improvement (9/15) as the intended application. The bioeconomy was viewed through a biotechnology lens by 23/83 studies, with 3/23 taking a valorisation perspective and 9/23 an end-of-life perspective, and the intended application was process improvement. Both the bioresource and biotechnology lens were used by 10/83 studies, but there was little consideration of the transition from fossil fuel economy to bioeconomy. Revenue streams were given more importance in these studies.

### Goal statement and the application

**Fig. 4:**
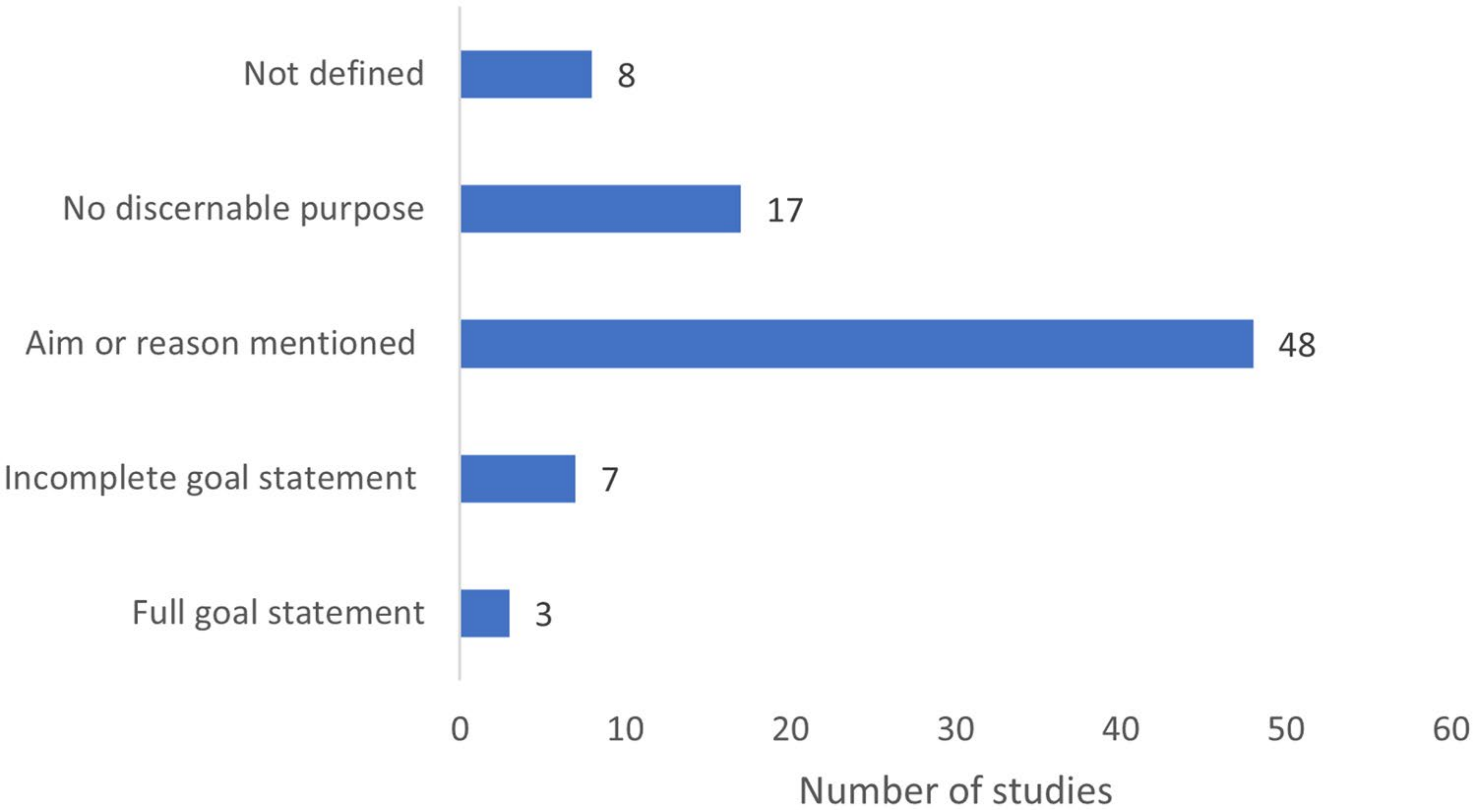
Completeness of the goal statements in the sample of 83 papers

Defining the goal is the first phase of any LCA as it defines the decision made for the scope and for all other phases of the LCA. The vast majority of studies did not define the goal clearly (Fig. 4). Only 3 studies presented a full goal statement as per the ISO guidelines, identifying all four components (reason, application, audience and stating if any comparative assertions for public disclosure), while another 7 addressed the reason and application only. The majority of the studies (48/83) described an aim or reason for the study in place of a formal goal statement, which might reflect the norms for scientific publishing but falls short of the requirements of an ISO standard LCA. Over 21% of the studies had no discernible purpose (17/83), while 8/83 did not refer to the goal because the overall purpose of the publication was a broader economic or techno-economic analysis and the necessary mandatory elements of the LCA part of the research were not described.

**Fig. 5:**
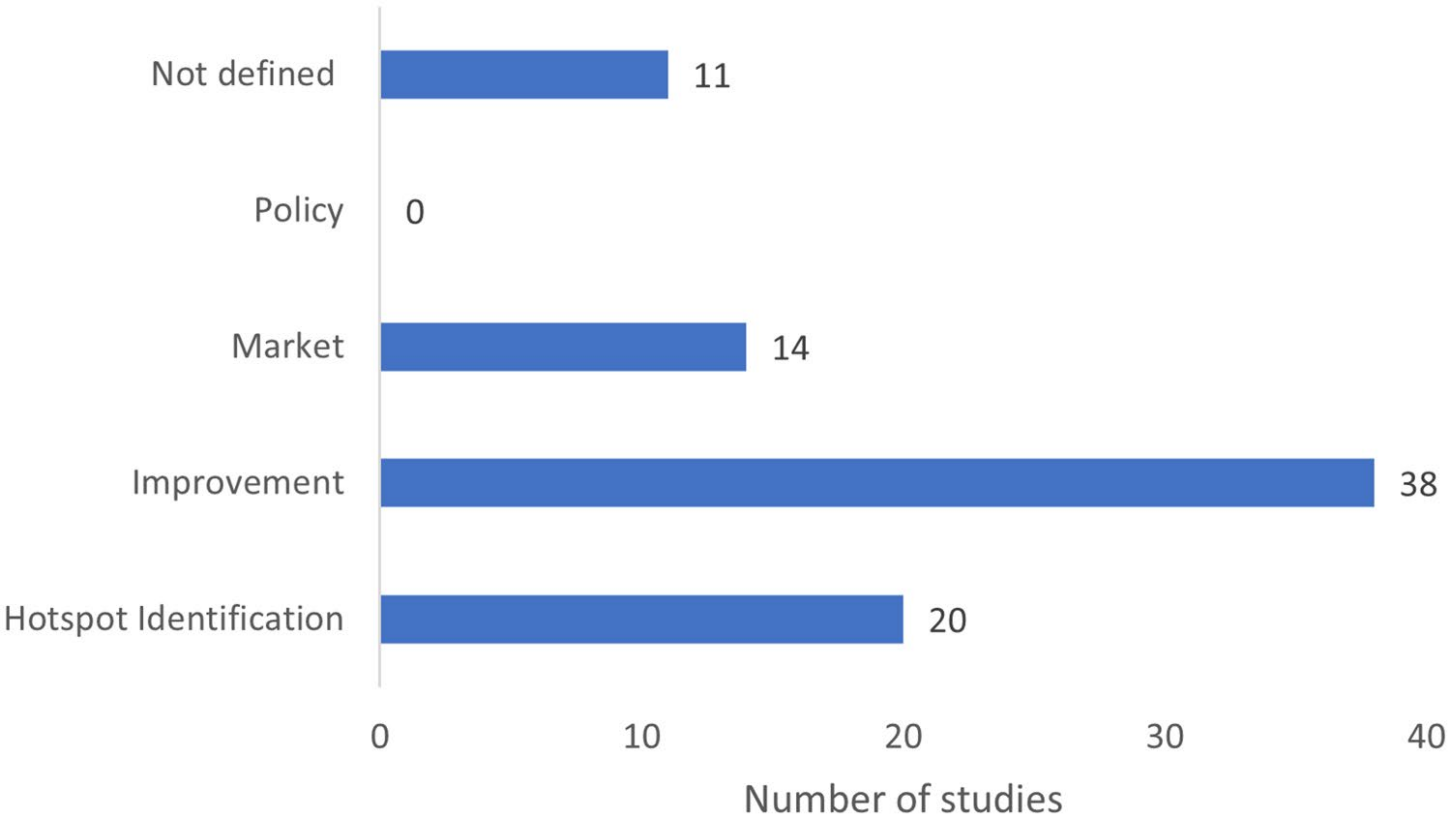
Intended application in the goal statement in the sample of 83 papers

The intended application, as clearly stated or inferred, indicated that the majority of studies were directed towards reducing environmental impact (Fig. 5), either by process improvement (38/83), hotspot identification (20/83) or as a basis for a business plan for developing a new market (14/83). Almost half of the studies aimed at process improvement considered novel feedstock (19/38) and how the efficiency could be improved. Most of the process improvement studies were done at a laboratory scale (33/38) and route to impact tended to be unclear as the goal statement did not address either the audience or the commissioner (21/33). The retrospective studies ALCA and CLCA indicated a focus on the process rather than the end product and its usability in the market. Of the 20 studies where hotspot analysis was the intended application, 15 had either identified or implied the commissioner. In these 15, the results were also communicated with the audience in order to take informed decisions based on the analysis (focussing on the process). None of the bioeconomy studies included was intended for policy development. Just under 13% of the studies (11/83) did not state or imply how the study might be used. It should be noted that the studies that presented a clear goal and scope opted for different life cycle approaches from one another such as consequential, hybrid and attributional. The common factor observed in the three studies was that they paid attention to the stakeholders involved and how the interpretations would impact the process in real time. This may also encourage stakeholders to act based on the results and interpretations obtained from the studies. For example, Moretti et al. (2020) studied the production of polypropylene from used cooking oil where the authors used attributional LCA to answer clear questions, which helped in producing robust results.

### Function and functional unit

**Fig. 6:**
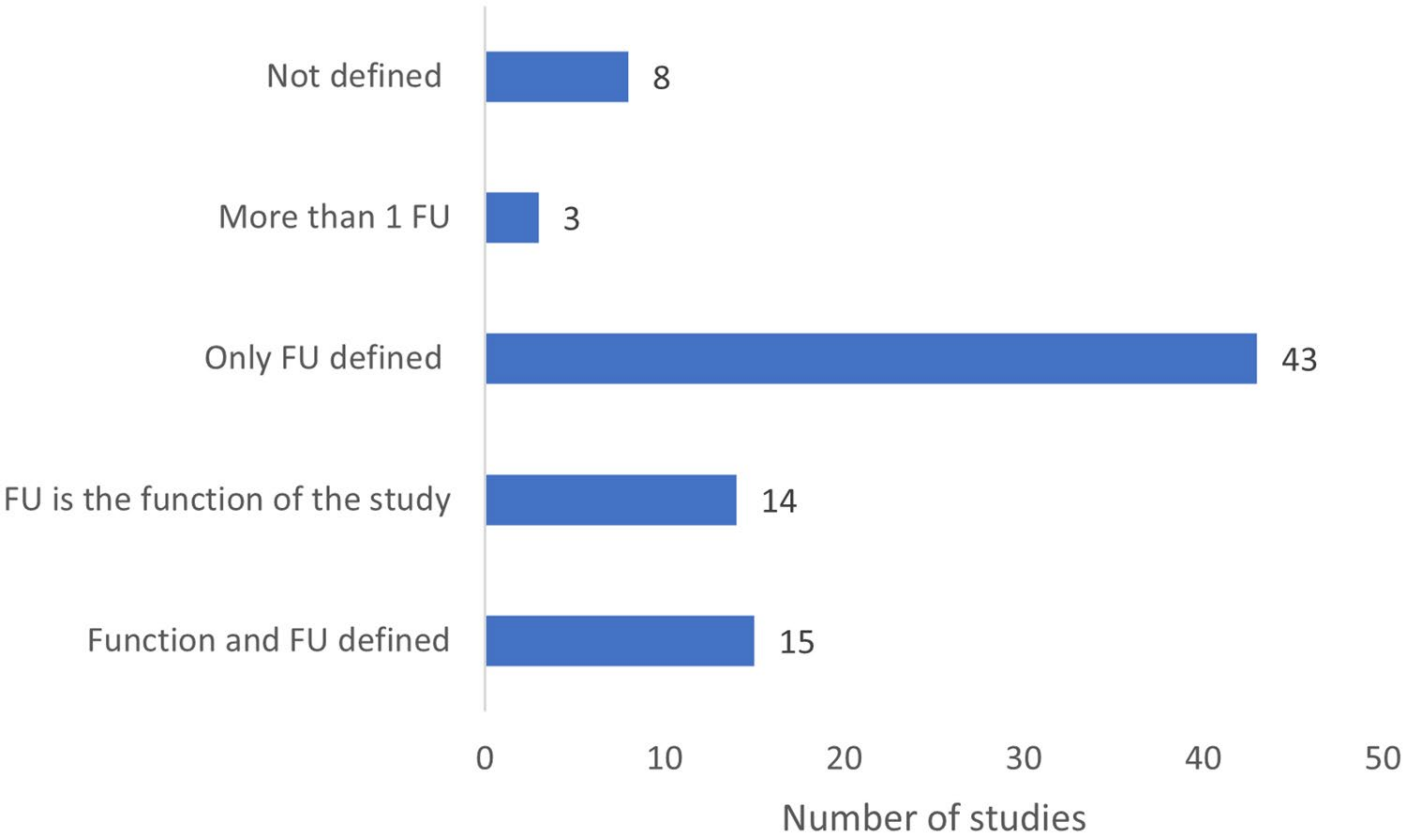
Function and functional unit presentation in the sample of 83 papers

To understand how practitioners have interpreted function in the context of the commissioner and previous studies, the function of the system needs clear definition closely related to the goal and the stakeholders involved. The functional unit (FU) is a quantitative measure combined (usually) with units to describe the function of the system. The FU cannot be independent of the activity of the system, which means that its magnitude should be proportional to the amount of activity happening in the system. Only 15/83 studies clearly defined both the function of the system and the functional unit chosen to represent the function, but a further 14/83 indicated that the FU was the function. Over half of the studies (43) only defined the FU, leaving it to the reader to work out what function was understood by the practitioner and/or commissioner. A small number (3/83) of the studies defined multiple functions and FU, while 8/83 did not define either. Overall, more than 85% of the studies failed to meet the requirement of the ISO standard to unequivocally present what was understood to be the function of the system and to present the working FU (Fig. 6).

### System boundary

**Fig. 7:**
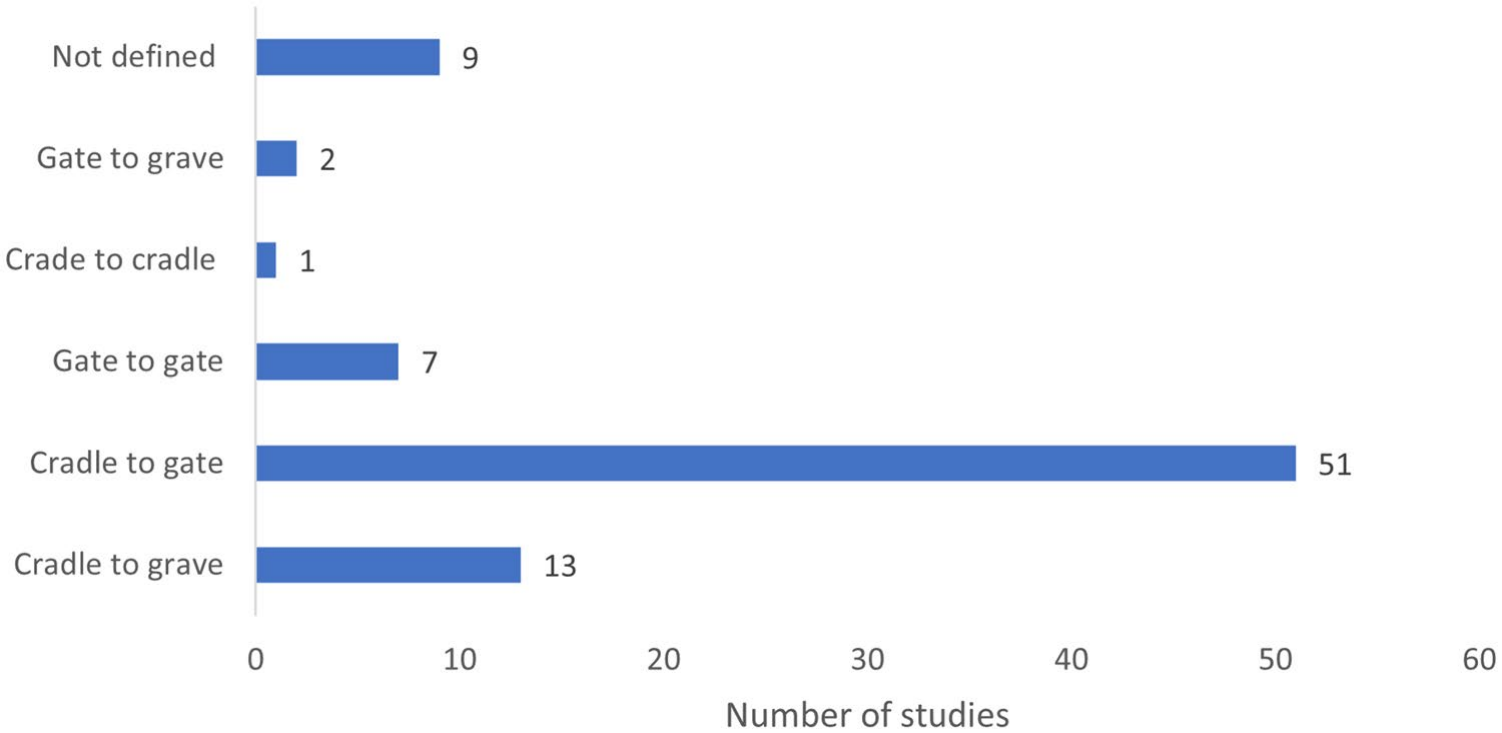
System boundary definitions in the sample of 83 papers

The definitions of bioeconomy presented in the introduction imply that systems delivering products, processes and services within the bioeconomy should be considered holistically, including all components from cradle to production through a cycle of use, reuse, recycling and recovery and ultimately to the end of life. The system boundary should include those processes that are part of the system being analysed. The studies reviewed were considered in terms of which linear or circular components were included in the LCA (Fig. 7). The majority of the studies (51/83) were from cradle to gate, meaning that the capture of feedstocks and some kind of process technology were involved, but no consideration was given to how the resulting materials would be used and its end of life. A gate-to-gate system boundary was used by 7/83 studies, and 2/83 were gate to grave, both focused on process technology. A cradle to grave system was used by 13/83 studies, and only 1 study considered a circular cradle to cradle system. Just over 10% of the studies (9/83) did not define the system boundary, and it was not possible to work out what the practitioners had used. Less than 18% of the studies defined a complete system that included raw material extraction, production, use and end-of-life stages, which aligns with the identified focus on process improvement and hotspot identification, but is perhaps less informative about the contribution to a sustainable bioeconomy, i.e. most studies were not focussed on the transition from fossil to bioeconomy.

### Impact categories and methods

**Fig. 8:**
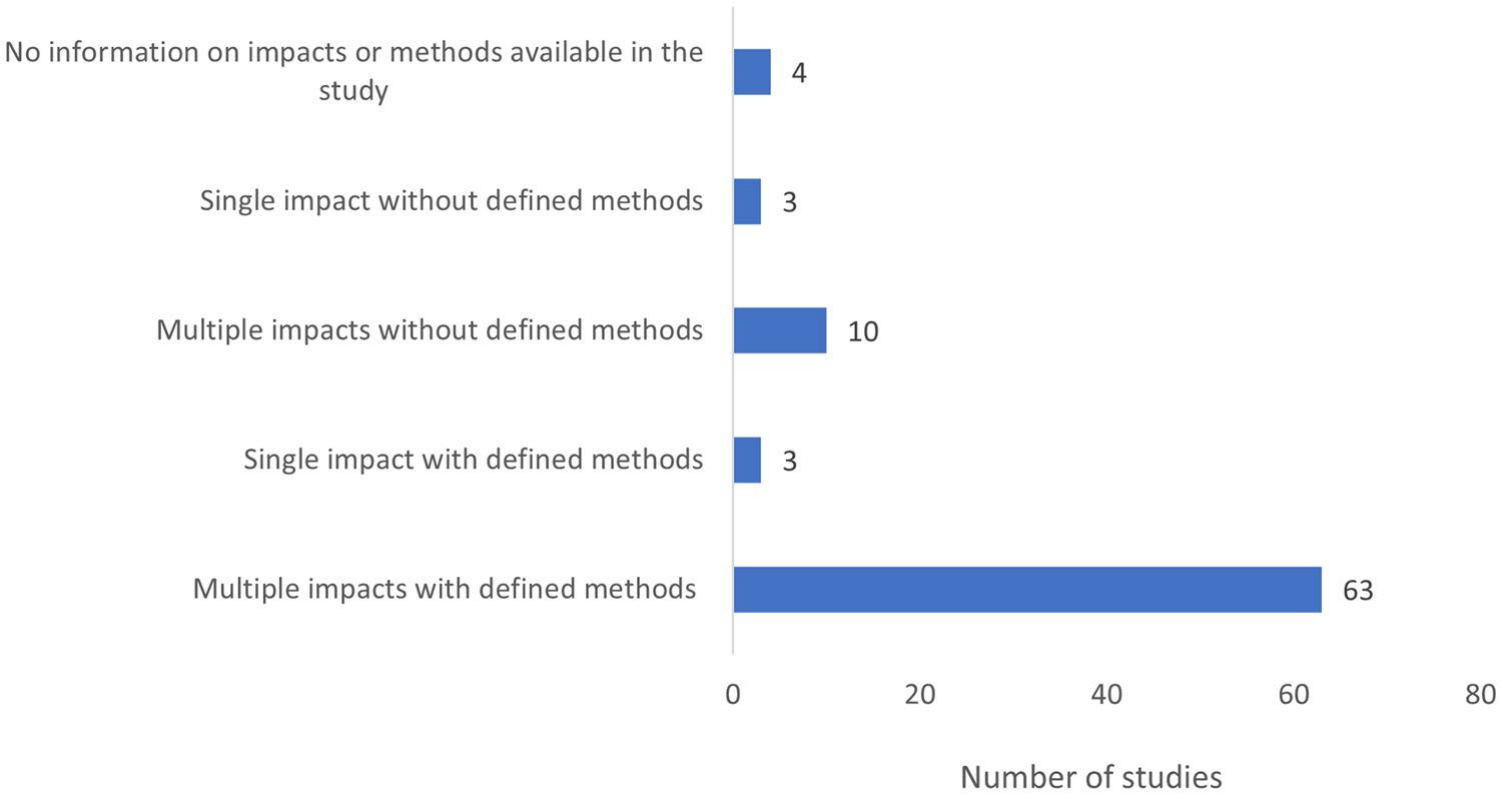
Use of impact methods and impact categories in the sample of 83 papers

The life cycle impact assessment is the phase where the elementary flows in the inventory are classified and characterised to express the environmental impacts of the system, as chosen during the scope stage. There are several impact methods available to use; however, there is no set reference method used by LCA practitioners, unless specific product category rules have been followed. ISO 14040 makes it mandatory for LCA practitioners to explicitly list the impact categories selected for the study and the impact methods used. The analysis (Fig. 8) showed that 4/83 of the studies made no mention of either the impact categories or the impact methods used. The impact methods were not mentioned by 13/83 studies, of which 3 studies considered a single impact category (climate change impact) and 10 studies considered multiple impact categories typically including climate change impact and fossil fuel depletion. Due to missing impact method detail, there is no clarity how the impact categories were calculated or the reason for selection. The majority of the studies defined both impact categories and methods (63), with 3 defining a single category related to climate change. The selection of impact categories in some studies appeared to be a purposeful, informed decision, whereas others seemed to merely calculate as many as possible.

**Fig. 9:**
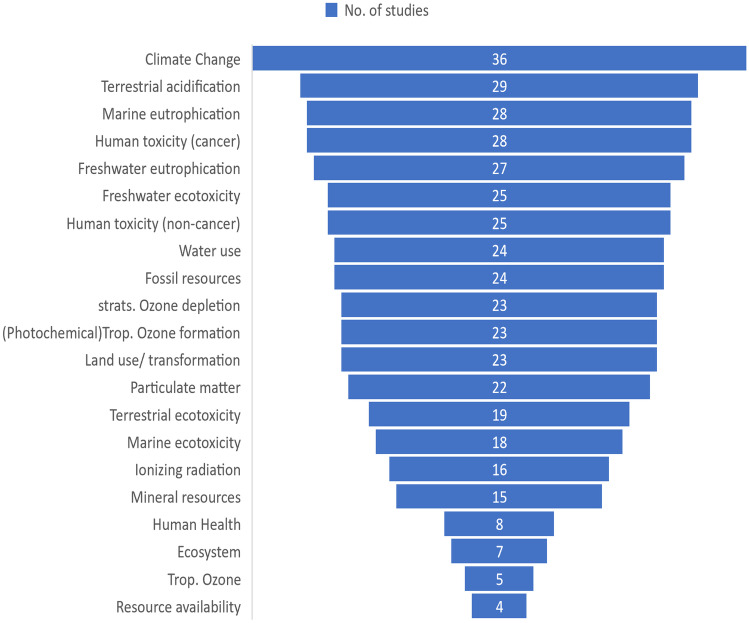
Number of studies using each impact categories from those studies that used the ReCiPe impact method

The impact methods mentioned in the 70/83 studies were ReCiPe (37/83), IPCC (1/83), TRACI 2 (4/83), CML (16/83), EDIP 2003 (1/83), ILCD 2011 (5/83), IMPACT 2002 + (3/83) and USETOX (3/83). ReCiPe was the most common impact method used, which is a merger of the midpoint CML method and the endpoint Eco-indicator 99 method (Matthews et al. 2014). Looking at the 37 studies that used the ReCiPe method, end point methods were only used by 4/37 studies. Figure 9 identifies most and least common impact categories for the 37 studies which used ReCiPe as their impact method. Climate change has clearly gained the most attention, followed by terrestrial acidification, eutrophication and ecotoxicity. The vast majority of studies have considered multiple impacts and have clearly defined the methods used to calculate these impacts, in compliance with ISO standard requirements. The majority of studies have focused on midpoint, rather than endpoint impacts.

### Interpretation

Life cycle interpretation is the final phase of an LCA study, where the results of LCI, LCIA or both are summarised and significant issues identified and discussed as a basis for drawing conclusions and making recommendations aimed at the target audience based on the goal statement (ILCD 2010).

**Fig. 10:**
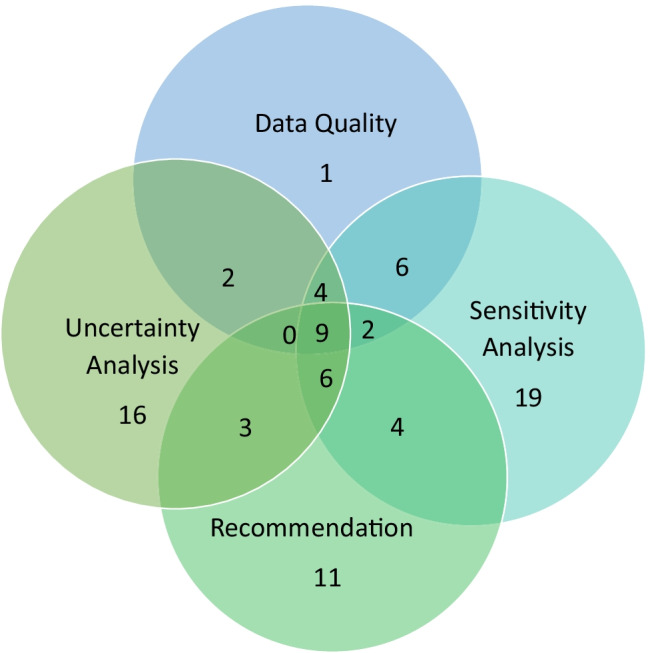
Interpretation and its components distributed in the studies (numbers represent the number of studies in each category)

The specific details of the recommendation and conclusion competent of the LCA interpretation were not analysed in detail, rather the focus was on the use of methods used to identify significant issues (sensitivity analysis, uncertainty evaluation, data quality) and whether explicit recommendations were made (Fig. 10). Sensitivity analysis was used in 50/83 studies and uncertainty analysis in 40/83 studies. Data quality was assessed in 24/83 studies. 35/83 of the studies made specific recommendations, but how useful these might have been for the intended application (where known) was not clear.

## Discussion

### Compliance of goal and scope with the ISO standard

This analysis suggests that the majority of the LCA studies published in the peer-reviewed literature from 2006 to 2021 were either (i) not in compliance with the ISO standards or (ii) space and style limitations of the publication process prevented competent practitioners from properly conveying their work. Lack of adherence to the ISO standard, or other rules based on the standard, does not mean that a study is not legitimate but does suggest a lack of understanding about why the standard was developed to include the requirements laid down. Compliance with the ISO standard is important for transparency, reproducibility and acceptance (Matthews et al. 2014; Santagata et al. 2021). It was recognised early in the development of LCA that there is much scope for bias (both intentional and unintentional) and unethical representation of a product, process or service (Cooper and Gutowski 2018). LCA cannot be subjected to empirical validation in the way used to provide confidence with mathematical models. The results of this study make it clear that those presenting bioeconomy LCA studies in the literature value the evaluation of the data quality (93% of the studies reviewed) and numerical calculation component of the interpretation (69% of the studies reviewed) far more than the transparent laying out of the rules by which the study was conducted during the goal and scope stage (13% of the studies reviewed). This is particularly important even if the work is not intended to make comparisons (where expert review should be included in the LCA method before publication), because informal comparisons are the norm for scientific publishing. Furthermore, if the work might be used as the basis for more specific product categories, such as for environmental product declarations or labels (International Council of Chemical Associations 2019), transparent presentation of the goal and scope is essential for confidence in the system.

The goal statement is the most important stage of any LCA study because it defines *why* it is being done and *how* it will be used. It effectively sets the terms and conditions of the work and offers a transparent view for the target audience. The ISO standard requires four definitive statements: (i) reason for carrying out the study, which should probably go beyond merely wanting to know the environmental impact of the product, process or service; (ii) the application, which should explain how the study will be used and sets the context for the conclusions and recommendations during the interpretation stage; (iii) the intended audience; and (iv) whether the results will be used for comparative assertions made to the public, which triggers methodological requirements during the later stages of the study. The International Reference Life Cycle Data System (ILCD 2010) goes beyond the ISO standard and suggests stating any methodological assumptions and impact limitations that might influence the interpretation and an unequivocal statement of about the commissioner and other influential actors. The integrity of a study requires these issues to be clearly stated so the audience (or journal reader) can understand the context of what they are reading. This review found the goal statement was incomplete in 96% of the studies. The results suggest that practitioners (and perhaps editors) working with LCA and the bioeconomy believe that conforming to the standard is perhaps desirable but not necessary. Muench and Guenther (2013) found from a systematic review of bioenergy LCA that methodological choices affect transparency and thus the value of recommendations (Muench and Guenther 2013). Suhariyanto et al. (2017) suggested that “defining a single goal statement is allowable as long as it is easily perceivable and there is not more than one interpretation”, but they also found that authors’ definition of a standard goal statement tends to be incorrect, incomplete and not in compliance with ISO standards (Suhariyanto et al. 2017). As the ISO (2006) standard is not restrictive in nature and applying it correctly maintains integrity by enhancing commissioner, practitioner and audience understanding, it is not clear why this situation has emerged.

The scope defines *what* system is being studied, *where* that system operates and *which* impacts are important. The function and the FU depend on the goal statement and the commissioning perspective, and in over 96% of the studies reviewed, this description was incomplete. There was a tendency not to clearly define the function of the system (82% of the studies reviewed), which means it is questionable whether the results can be used in the context of scientific research (e.g. simple comparisons), because the reader has to guess what the practitioner believed to be the system function, and cannot judge whether the FU properly captures the commissioning perspective (Oldfield et al. 2018). It is imperative for an LCA study to report the function, which will help in identifying the functional unit (Rønning and Brekke 2014). Absence of a clear definition of the system boundary also makes the LCA ambiguous and non-transparent (Martínez-Blanco et al. 2015). Almost 11% of the studies reviewed did not define the system boundary, and this was associated with an incomplete goal statement. Only 18% of the studies reviewed considered a full life cycle from cradle to grave (with none taking a circular perspective). The omission of life cycle stages only makes sense if it does not significantly limit the conclusions and recommendations required for the stated application. Almost 70% of the studies reviewed assumed that downstream use would have no impact on the recommendations, perhaps because there was a focus on hotspots (24%) and process improvement (45%) (a bio-technological/bioresource view) rather than the overall impact in a bioeconomy context (a bio-ecological view). Just under 30% of the studies reviewed considered the end-of-life stage, with these tending to take a valorisation perspective (i.e. waste management, 58%). This neglects environmental burden of feedstocks supplying the value chain (the zero burden assumption) and probably overestimates successful achievement in the bioeconomy. It is clear that most LCA studies conducted to date offer little real insight into the contribution of a technology in the context of the system in which it is deployed to creating a sustainable bioeconomy. The focus has been on merely being better than fossil, linear economy. Since the majority of the goal statements (96%) only mention the aim or the objective of the study or did not define the study, the quality of the goal and scope statements is so poor that it is not possible to understand perspective in many cases. It is clear that the use of LCA in a sustainable bioeconomy context (Ministry of Infrastructure and the Environment 2011) will require greater attention to the goal and scope, both in practice and publication. The value and integrity of the literature seem to be undermined by not rigorously addressing the first and most important stage of an LCA study.

All bioeconomy related LCA studies published in the scientific literature in the future should include the final goal statement either used by the practitioner or agreed by the practitioner and commissioner partnership that provides the context for the LCI, LCIA and interpretation presented. This might be separate from the *research objective*, as the two need not be the same. A complete scope statement should also be available to readers either in the main text or as supplementary materials. This is essential for transparent use of LCA, regardless of the intended application, for the integrity of the research being published by the relevant scientific journals.

### Actor and stakeholder views of the bioeconomy in LCA studies

Bioeconomy is important for business, the economy, governments, policy makers and researchers, and it is now argued that it is a key part of the solution for transition to a sustainable future (Urmetzer et al. 2020). Despite this, there seems to be a significant gap between the perspectives of the commissioners of the studies reviewed and the bioeconomy policies that caused the studies to be conducted, mainly due to incomplete goal and scope statements.

Most of the LCA studies reported were conducted in Europe where the main perspective was waste management. Off these, nearly 90% viewed the bioeconomy through a bioresource lens. This is perhaps due to increased numbers of funding programmes made available to strengthen the bio-based economy in Europe (Bio-based Industries 2014). The USA was the source of only 8.5% of the bio-based LCA studies, but these had an equal view through both the bioresource and biotechnology lenses. This perhaps reflects the changing understanding of the policies that strengthen the bioeconomy due to the close link between funding and the changing political administrations (The International Advisory Council on Global Bioeconomy 2020).

The commissioning perspective is important because it places the goal and scope in context and enables transparency for the audience. The number of reviewed studies that did not make the commissioning perspective clear might reflect the increasingly common use of LCA as a mandatory component of research projects (Bio-based Industries 2014). In this case, there is no explicit commissioner, but there is a clear perspective, which is to use LCA to provide evidence to support or justify investment of public funds (2017). Almost 27% of the studies viewed bioeconomy through a biotechnology lens to identify process improvement, and there was only 13% overlap with the valorisation perspective. These studies focused on increasing efficiency and profit, with reducing environmental impact being no more than an added advantage. The studies that viewed bioeconomy market application through a biotechnology lens focussed on commercial upscaling of technology under the pretence of transition towards bioeconomy, but half did not define a system boundary, and none viewed bioeconomy through a bio-ecological lens. The bioresource lens (50%) was a common way to view bioeconomy for LCA studies, focussing on novel feedstock in the context of process improvement or hotspot analysis.

All bioeconomy LCA studies should report the commissioning perspective. The ISO standard requires the commissioner’s details to be recorded on the report, which does not translate well to scientific publication, and the commissioner is a part of the critical review, which is an optional element. Building on the (ILCD 2010) guidelines, the commissioner’s perspective should be explicitly stated in the goal statement and for journal papers should be in either the main text or supplementary materials.

### Contribution to understanding transformation to bioeconomy

There are a number of challenges that need to be overcome to properly integrate LCA into innovation and development in order to better understand transition to bioeconomy. Prospective environmental assessment of novel technologies and processes is potentially important for designers and engineers. LCA can be used by policy makers and those associated with scientific grants in order to fund projects and nudge manufacturers towards sustainable applications (Cooper and Gutowski 2018). At early stages in the innovation cycle, the shortcomings of LCA should be recognised, but if the main purpose of the study is to understand contribution of hotspots or to realise technology specific optimization and potential for improvement, then an attributional life cycle approach can be employed (Rønning and Brekke 2014). This has been the focus of the majority of the bioeconomy studies reported in the literature to date. To understand transition to bioeconomy, Cucurachi et al. (2018) noted that identifying incumbent technology, best available technology performing the same function and overall system functionality is an important challenge that must be addressed. A major shortcoming noted in the studies analysed here was that most did not consider the industrial symbiosis needed between feedstock, technology, primary products, side streams, downstream valorisation and long-term circularity in order to properly understand the transition pathways required. A consequential approach should be adopted when an important decision needs to be made with respect to either a business plan or adherence to governmental policies (Bergerson et al. 2020). There is a significant challenge in establishing a robust means of thinking holistically when a technology or system is at an early innovation stage. The implications of trade-offs are also important, such as cost vs. energy demand under current and future pricing. The results reported here show that most studies identified the main cause of impacts and presented conclusions (51%) without the context of the downstream components of the system. In other words, the bioeconomy technologies were imagined as displacers for feedstocks and processes to adapt business as usual, rather than transformers of the system to a sustainable footing. Furthermore, recommendations were not provided by all studies (42%), which prevents the commissioner from acting on the LCA results. Even when a recommendation was given, the type of recommendations did not necessarily aid in the decision-making process either because the data were secondary (17% overlap) or the system was not developed sufficiently to be confident about the data (71%), a problem that could be solved using distributions rather than point values. Current sectoral rules (e.g. product category rules) tend to focus on retrospective, business-as-usual situations, so they are not suitable for bioeconomy transition studies. Frameworks need to be formulated to characterise and classify technologies and scale up methods and to provide guidelines for the environmental assessment of emerging technologies in the context of transition to bioeconomy. The first step in promoting prospective environmental LCA is to acknowledge and ensure that LCA is conducted holistically and if the interpretation will play a role in decision-making about an emerging technology or process (McManus and Taylor 2015). The body of studies in the literature is not a strong foundation for this purpose.

Use of consequential LCA becomes appropriate once a technology or system has been innovated to pilot or preliminary application scale. At this stage, it should be possible to envisage the place of the materials and products in the market, and there should be sufficient business intelligence to specify the wider system into which the innovation fits. Rehl et al. (2012) compared the same biogas system, using attributional and consequential LCA, and found major differences in the conclusions drawn, and from this, it was concluded that it is essential to exactly define the goal and scope of the study, thus making sure that the right methodological approach is chosen (Rehl et al. 2012). While most studies reported in the literature to date have value for identifying possible problems and perhaps solutions, they do not offer necessary insight to understand transition to bioeconomy. It is important for stakeholders to understand the attributes of bioeconomy that needs to change from the business as usual perspective (Hakovirta et al. 2020).

The data collected showed that only three studies presented a full goal statement compliant with ISO standards. The analysis implies that the LCA studies published in the last 15 years will not be all that useful for managing the transition to bioeconomy. In order for LCA to develop effectively and be used by and for policy makers, researchers and stakeholders, investment is required from both the industrial and the government sectors (McManus and Taylor 2015). Investing in four main areas can support the expansion of LCA and maintain its credibility and reliability. These are (1) greater integration between the attributional and consequential practice, ensuring that the right method is used for a particular goal, (2) developing transparent mechanisms to convey uncertainty and comparability and drawing conclusions appropriately, (3) greater understanding and integration of the industrial symbiosis and downstream uses of biomaterials and feedback mechanisms and (4) data to fill gaps.

### Contribution to understanding sustainable bioeconomy

Conventional environmental LCA as applied to bioeconomy resources and technologies over the last 15 years will not provide sufficient information to allow an assessment of the sustainability of bioeconomy, since they have not addressed the system holistically and have not considered difficult questions such as fair share of resources (Wohlfahrt et al. 2019), rates of exploitation that are renewable, environmental thresholds and buffering capacity (D’Amato et al. 2020), balanced consumption (El-Chichakli, 2016) and circularity (European Commission 2014). The transition to bioeconomy requires more than simply replacing the fossil fuels; it is a complex and irreversible adaptation of the whole system, which involves innovation, new lifestyles and changes to governance (Pyka et al. 2022). Life cycle assessment modelling choices will play an important role in the studies that provide quantitative evidence for managing and navigating the transition to bioeconomy, but historical studies are not that valuable because of the choices made.

At an even more basic level, the selection of impact categories and method has been quite broad (Fig. 8) and does not necessarily offer great insight into the changing status of natural capital, despite suitable methods having been developed in the early period covered by the review (Zhang et al. 2010). The lack of detail about specific methods in the studies analysed might be explained by the ISO standard stating “mandatory selection of impact categories” not impact methods, but it has long been recognised that the choice of method influences the conclusions that can be drawn (Matthews et al. 2014; Bergerson et al. 2020). The studies reviewed made little reference to the reason for choosing particular impact categories. The ISO standard requires the impact assessment to consist of “comprehensive set of environmental issues”, to avoid limiting scope of the study. Recent bioeconomy LCA studies have considered a large number of impact categories, but the reasons for inclusion or exclusion in each study were not necessarily explained. In the future, more attention should be paid to purposeful selection of impact categories (Esnouf et al. 2019) and standardisation of impact methods to ensure comparability of studies. If LCA studies are going to provide meaningful information for actors and stakeholders to assess whether a system will be able to operate sustainably, a new approach will be required that goes beyond mere eco-efficiency or doing less harm as these are not necessarily indicative of sustainability.

## Conclusions

The LCA standard was formalised by the ISO Technical Committee (TC 207), sub-committee SC 5 in Paris in November 1993. This standard was inspired by the Code of Practice developed by the Society of Environmental Toxicology and Chemistry (SETAC). The need for a standard emerged from the growing understanding that LCA could become a useful methodological tool for identifying environmental aspects if it was used consistently and transparently (Sven-Olof Ryding 1997). There were many critiques of pre-2006 (ISO 14040) studies that did not address uncertainty analysis, weighting, allocation transparency and other methodological issues (Pryshlakivsky and Searcy 2013). The review of 83 LCA studies related to bioeconomy revealed that the reporting in the scientific literature does not present sufficient detail, particularly of the goal and scope, to ensure the integrity of the research. In the future, all LCA reports related to bioeconomy should include a complete goal and scope statement, whether in the main text or supplementary materials. The commissioning stakeholder perspective should also be presented. This will also result in a better understanding of how the bioeconomy is viewed. None of the studies reviewed looked at bioeconomy through a bioecology lens, perhaps reflecting a lack of holistic understanding of bioeconomy and a focus on incremental change to business as usual, rather than sustainable transition. These shortcomings affect the robustness and meaning of the LCA results and will have an impact on transition to bioeconomy. Thus, complying with the ISO standard is a minimum requirement to conduct a comprehensive LCA study. The majority of studies published to date offer little insight into the current status of bioeconomy, i.e. whether it is sustainable or not, and concepts of extended linearity, cascades and circularity, while present in the theoretical literature, have not entered mainstream practice. This means that these studies offer little meaningful insight into where transition to bioeconomy will fall short in terms of sustainable societies. A conceptual framework is required for conducting an efficient LCA study for emerging technologies or novel processes. In conclusion, the answer to the question, “is LCA being used in the best possible manner in the context of the emerging bioeconomy?” is no. As a result of this work, it can be recommended that methodological choices are given more attention, particularly, providing a complete goal and scope statement for all studies published, explaining the reasons for choices so the comparison of studies is easier, considering the role of circularity and ensuring data quality and uncertainty are highlighted. It is also recommended that studies address the system holistically, rather than truncating around the core technology. To do this, the study should encompass the core technology (the biotechnology lens), the necessary resources (the bioresource lens) and the wider environmental implications (the bioecology lens). Sustainable transition will require information about all three, but this is lacking in the studies published to date.

## Annex A

**Table:** 

S. No	Title	Year	Country	References	Journal
1	Bioresource utilisation by sustainable technologies in new value-added biorefinery concepts—Two case studies from food and forest industry	2013	Sweden	Ekman et al. (2013)	*Journal of Cleaner Production*
2	Life cycle assessment of commodity chemical production from forest residue via fast pyrolysis	2014	USA	Zhang et al. (2014)	*International Journal of Life Cycle Assessment*
3	LCA of 1,4-butanediol produced via direct fermentation of sugars from wheat straw feedstock within a territorial biorefinery	2016	Italy	Forte et al. (2016)	Materials
4	Uncertainty in the Life Cycle Greenhouse Gas Emissions from U.S. Production of Three Biobased Polymer Families	2016	USA	Posen et al. (2016)	*Environmental Science and Technology*
5	Climate Change Mitigation Challenge for Wood Utilization-The Case of Finland	2016	Finland	Soimakallio et al. (2016)	*Environmental Science and Technology*
6	Bioextraction potential of seaweed in Denmark—An instrument for circular nutrient management	2016	Denmark	Seghetta et al. (2016)	*Science of the Total Environment*
7	An environmental analysis of options for utilising wasted food and food residue	2016	Ireland	Oldfield et al. (2016)	*Journal of Environmental Management*
8	Material flow and sustainability analyses of biorefining of municipal solid waste	2017	UK	Sadhukhan and Martinez-Hernandez (2017)	*Bioresource Technology*
9	Life cycle assessment of orange peel waste management	2017	Italy	Negro et al. (2017)	*Resources, Conservation and Recycling*
10	Life cycle assessment of wood-plastic composites: Analysing alternative materials and identifying an environmental sound end-of-life option	2017	Germany	Sommerhuber et al. (2017)	*Resources, Conservation and Recycling*
11	Multi-product biorefineries from lignocelluloses: A pathway to revitalisation of the sugar industry?	2017	South Africa	Farzad et al. (2017)	*Biotechnology for Biofuels*
12	Seaweed as innovative feedstock for energy and feed – Evaluating the impacts through a Life Cycle Assessment	2017	Italy	Seghetta et al. (2017)	*Journal of Cleaner Production*
13	Novel miscanthus germplasm-based value chains: A life cycle assessment	2017	UK	Wagner et al. (2017)	*Frontiers in Plant Science*
14	Environmental performance of manure co-digestion with natural and cultivated grass – A consequential life cycle assessment	2017	Estonia and Poland	Pehme et al. (2017)	*Journal of Cleaner Production*
15	Climate-change and health effects of using rice husk for biochar-compost: Comparing three pyrolysis systems	2017	North Vietnam	Mohammadi et al. (2017)	*Journal of Cleaner Production*
16	Multi-criteria analysis of a biorefinery for co-production of lactic acid and ethanol from sugarcane lignocellulose	2017	South Africa	Mandegari et al. 2017)	*Biofuels, Bioproducts and Biorefineries*
17	Explorative environmental life cycle assessment for system design of seaweed cultivation and drying	2017	Netherlands	van Oirschot et al. (2017)	*Algal Research*
18	Life cycle assessment of feedstock supply systems for cellulosic biorefineries using corn stover transported in conventional bale and densified pellet formats	2017	USA	Manandhar and Shah (2017)	*Journal of Cleaner Production*
19	Environmental impacts of producing bioethanol and biobased lactic acid from standalone and integrated biorefineries using a consequential and an attributional life cycle assessment approach	2017	Denmark	Parajuli et al. (2017)	*Science of Total Environment*
20	A life cycle assessment of poly-hydroxybutyrate extraction from microbial biomass using dimethyl carbonate	2017	Italy	Righi et al. (2017)	*Journal of Cleaner Production*
21	Uncertainties in corn stover feedstock supply logistics cost and life-cycle greenhouse gas emissions for butanol production	2017	USA	Baral et al. (2017)	*Applied Energy*
22	Bio-electrochemical conversion of industrial wastewater-COD combined with downstream methanol synthesis-an economic and life cycle assessment	2018	Germany	Streeck et al. (2018)	*Green Chemistry*
23	The implications of stakeholder perspective for LCA of wasted food and green waste	2018	Ireland	Oldfield et al. (2018)	*Journal of Cleaner Production*
24	Environmental assessment of biorefinery processes for the valorization of lignocellulosic wastes into oligosaccharides	2018	Spain	Gonzalez-Garcia et al. (2018)	*Journal of Cleaner Production*
25	Choice of mineral fertilizer substitution principle strongly influences LCA environmental benefits of nutrient cycling in the agri-food system	2018	Norway	Hanserud et al. (2018)	*Science of Total Environment*
26	Eco-efficiency assessment of bioplastics production systems and end-of-life options	2018	Thailand	Changwichan et al. (2018)	*Sustainability (Switzerland)*
27	Life cycle assessments of bio-based sustainable polylimonene carbonate production processes	2018	UK	Zhang, del Rio-Chanona, Wagner et al. (2018b)	*Sustainable Production and Consumption*
28	Life cycle, techno-economic and dynamic simulation assessment of bioelectrochemical systems: A case of formic acid synthesis	2018	UK	Shemfe et al. (2018)	*Bioresource Technology*
29	Comparative environmental Life Cycle Assessment of integral revalorization of vine shoots from a biorefinery perspective	2018	Spain	Gullón et al. (2018)	*Science of Total Environment*
30	Life cycle assessments for biomass derived sustainable biopolymer & energy co-generation	2018	UK	Zhang, del Rio-Chanona and Shah (2018a)	*Sustainable Production and Consumption*
31	An environmental and economic analysis of the wood-pellet chain: two case studies in Southern Italy	2018	Italy	Pergola et al. (2018)	*International Journal of Life Cycle Assessment*
32	From wood to resin-identifying sustainability levers through hotspotting lignin valorisation pathways	2018	Austria	Lettner et al. (2018)	*Sustainability (Switzerland)*
33	Gate-to-gate life cycle assessment of biosurfactants and bioplasticizers production via biotechnological exploitation of fats and waste oils	2018	UK	Kopsahelis et al. (2018)	*Journal of Chemical and Biotechnology*
34	Life-cycle assessment on food waste valorisation to value-added products	2018	Hong Kong	Lam et al. (2018)	*Journal of Cleaner Production*
35	The future of Swedish food waste: An environmental assessment of existing and prospective valorization techniques	2018	Sweden	Brunklaus et al. (2018)	*Journal of Cleaner Production*
36	Revealing the Environmental Advantages of Industrial Symbiosis in Wood-Based Bioeconomy Networks: An Assessment From a Life Cycle Perspective	2018	Germany	Hildebrandt (2018)	*Journal of Industrial Ecology*
37	Scale-up and Sustainability Evaluation of Biopolymer Production from Citrus Waste Offering Carbon Capture and Utilisation Pathway	2019	UK	Durkin et al. (2019)	*Chemistry*
38	Competitive use of sugarcane for food, fuel, and biochemical through the environmental and economic factors	2019	Thailand	Silalertruksa and Gheewala (2019)	*International Journal of Life Cycle Assessment*
39	Environmental sustainability assessment of HMF and FDCA production from lignocellulosic biomass through life cycle assessment (LCA)	2019	Spain	Bello et al. (2019)	*De Gruyter*
40	Assessing the technical and environmental performance of wood-based fiber laminates with lignin based phenolic resin systems	2019	Germany	Hildebrandt et al. (2019)	*Resources, Conservation and Recycling*
41	Comparative life cycle assessment of first- and second-generation ethanol from sugarcane in Brazil	2018	Brazil	Maga et al. (2019)	*International Journal of Life Cycle Assessment*
42	Sustainability of carbon delivery to an algal biorefinery: A techno-economic and life-cycle assessment	2019	USA	Somers and Quinn (2019)	*Journal of CO2 Utilization*
43	Eco-efficiency analysis of recycling recovered solid wood from construction into laminated timber products	2019	Germany	Risse et al. (2019)	*Science of Total Environment*
44	Life Cycle Assessment of waste disposal from olive oil production: Anaerobic digestion and conventional disposal on soil	2019	Italy	Batuecas et al. (2019)	*Journal of Environmental Management*
45	Maximizing environmental impact savings potential through innovative biorefinery alternatives: An application of the TM-LCA framework for regional scale impact assessment	2019	Denmark	Vega et al. (2019)	*Sustainability (Switzerland)*
46	Integrated evaluation of wine lees valorization to produce value-added products	2019	Spain	Cortés et al. (2019)	*Waste Management*
47	Sustainability and life cycle assessment (LCA) of macroalgae-derived single cell oils	2019	UK	Parsons et al. (2019)	*Journal of Cleaner Production*
48	Assessing the environmental sustainability of glucose from wheat as a fermentation feedstock	2019	Spain (9 countries)	Salim et al. (2019)	*Journal of Environmental Management*
49	Life Cycle Impact Assessment of Polylactic Acid (PLA) Produced from Sugarcane in Thailand	2019	Thailand	Morão and de Bie (2019)	*Journal of Polymer and the Environment*
50	Process of fruit peel waste biorefinery: a case study of citrus waste biorefinery, its environmental impacts and recommendations	2019	India	Joglekar et al. (2019)	*Environmental Science and Pollution Research*
51	Environmental life cycle assessment of different biorefinery platforms valorizing municipal solid waste to bioenergy, microbial protein, lactic and succinic acid	2020	Copenhagen	Khoshnevisan et al. (2020)	*Renewable and Sustainable Energy Reviews*
52	Environmental impact assessments of integrated food and non-food production systems in Italy and Denmark	2020	Italy and Denmark	Lehmann et al. (2020)	*Energies*
53	Environmental hotspots of lactic acid production systems	2020	Denmark	Ögmundarson et al. (2020)	*Bioenergy*
54	Bio-combustion of petroleum coke: The process integration with photobioreactors. Part II – Sustainability metrics and bioeconomy	2020	Brazil	Severo et al. (2020)	*Chemical Engineering Science*
55	Life cycle assessment of giant Miscanthus: Production on marginal soil with various fertilisation treatments	2020	Poland	Krzyżaniak et al. (2020)	*Energies*
56	Life cycle assessment of anaerobic digestion of pig manure coupled with different digestate treatment technologies	2020	China	Duan et al. (2020)	*Environment International*
57	Life cycle environmental impact assessment of biomass materials in Japan	2020	Japan	Dente et al. (2020)	*Journal of Cleaner Production*
58	Life cycle environmental sustainability of valorisation routes for spent coffee grounds: From waste to resources	2020	UK	Schmidt Rivera et al. (2020)	*Resources, Conservation and Recycling*
59	Comparative life cycle assessment of microalgae cultivation for non-energy purposes using different carbon dioxide sources	2020	Italy	Porcelli et al. (2020)	*Science of Total Environmental*
60	Life Cycle Assessment of vegetable oil based polyols for polyurethane production	2020	Latvia	Fridrihsone et al. (2020)	*Journal of Cleaner Production*
61	Hybrid life cycle assessment of potato pulp valorisation in biocomposite production	2020	Italy	Chen et al. (2020)	*Journal of Cleaner Production*
62	Life cycle assessment of bagasse fiber reinforced biocomposites	2020	Brazil	Ita-Nagy et al. (2020)	*Science of Total Environmental*
63	Environmental life cycle assessment of polypropylene made from used cooking oil	2020	Netherlands	Moretti et al. (2020)	*Resources, Conservation and Recycling*
64	Upgrading wineries to biorefineries within a Circular Economy perspective: An Italian case study	2021	Italy	Ncube et al. (2021)	*Science of The Total Environment*
65	Cradle-to-grave life cycle assessment of single-use cups made from PLA, PP and PET	2021	Denmark	Moretti et al. (2021)	*Resources, Conservation and Recycling*
66	Novel insights in dimethyl carbonate-based extraction of polyhydroxybutyrate (PHB)	2021	Italy	Mongili et al. (2021)	*Biotechnology Biofuels*
67	Environmental life cycle assessment of cascade valorisation strategies of South African macroalga Ecklonia maxima using green extraction technologies	2021	South Africa	Zhang et al. (2021)	*Algal Research*
68	Circular economy in the agro-industry: Integrated environmental assessment of dairy products	2021	Italy	Oliveira et al. (2021)	*Renewable and Sustainable Energy Reviews*
69	Life cycle assessment of hetero- and phototrophic as well as combined cultivations of Galdieria sulphuraria	2021	Germany	Thielemann et al. (2021)	*Bioresource Technology*
70	Mitigating environmental impacts of milk production via integrated maize silage planting and dairy cow breeding system: A case study in China	2021	China	Huang et al. (2021)	*Journal of Cleaner Production*
71	Environmental impacts of protein-production from farmed seaweed: Comparison of possible scenarios in Norway	2021	Norway	Koesling et al. (2021)	*Journal of Cleaner Production*
72	Life cycle assessment of fish oil substitute produced by microalgae using food waste	2021	Germany	Bartek et al. (2021)	*Sustainable Production and Consumption*
73	Environmental performance of miscanthus-lime lightweight concrete using life cycle assessment: Application in external wall assemblies	2021	UK	Ntimugura et al. (2021)	*Sustainable Materials and Technologies*
74	Life cycle assessment of bacterial cellulose production	2021	Portugal	Forte et al. (2021)	*The International Journal of Life Cycle Assessment*
75	Life cycle analysis of fermentative production of succinic acid from bread waste	2021	Hong Kong	Gadkari et al. (2021)	*Waste Management*
76	Using life cycle assessment to quantify the environmental benefit of upcycling vine shoots as fillers in biocomposite packaging materials	2020	France	David et al. (2020)	*The International Journal of Life Cycle Assessment*
77	Early-stage sustainability assessment of enzyme production in the framework of lignocellulosic biorefinery	2020	Spain	Bello et al. (2021)	*Journal of Cleaner Production*
78	Life Cycle Assessment of Total Fatty Acid (TFA) Production from Microalgae Nannochloropsis oceanica at Different Sites and Under Different Sustainability Scenarios	2021	Italy	Gaber and Rosch (2021)	*BioEnergy Research 2021*
79	Comparative life cycle assessment of cellulose nanofibres production routes from virgin and recycled raw materials	2021	Italy	Gallo Stampino et al. (2021)	*Molecules*
80	Wastewater treatment using oxygenic photogranule-based process has lower environmental impact than conventional activated sludge process	2021	France	Brockmann et al. (2021)	*Bioresource Technology*
81	How sustainable are biopolymers? Findings from a life cycle assessment of polyhydroxyalkanoate production from rapeseed-oil derivatives	2020	Poland	Nitkiewicz et al. (2020)	*Science of the Total Environment*
82	Evaluation of the life cycle of an automotive component produced from biocomposite	2020	Canada	Roy et al. (2020)	*Journal of Cleaner Production*
83	An attributional life cycle assessment of microbial protein production: A case study on using hydrogen-oxidizing bacteria	2021	Finland	Järviö et al. (2021)	*Science of The Total Environment*

## Supplementary information

Below is the link to the electronic supplementary material.

Supplementary file1 (XLSX 40 KB)

## References

[CR1] Bach V, Lehmann A, Görmer M, Finkbeiner M (2018). Product environmental footprint (PEF) pilot phase—comparability over flexibility?. Sustainability.

[CR2] Baral NR, Quiroz-Arita C, Bradley TH (2017). Uncertainties in corn stover feedstock supply logistics cost and life-cycle greenhouse gas emissions for butanol production. Appl Energy.

[CR3] Bartek L, Strid I, Henryson K (2021). Life cycle assessment of fish oil substitute produced by microalgae using food waste. Sustain Prod Consum.

[CR4] Batuecas E, Tommasi T, Battista F (2019). Life cycle assessment of waste disposal from olive oil production: anaerobic digestion and conventional disposal on soil. J Environ Manage.

[CR5] Bello S, Pérez N, Kiebist J (2021). Early-stage sustainability assessment of enzyme production in the framework of lignocellulosic biorefinery. J Clean Prod.

[CR6] Bello S, Salim I, Méndez-trelles P (2019). Environmental Sustainability Assessment of HMF and FDCA Production from Lignocellulosic Biomass through Life Cycle Assessment ( LCA ).

[CR7] Bergerson JA, Brandt A, Cresko J (2020). Life cycle assessment of emerging technologies: evaluation techniques at different stages of market and technical maturity. J Ind Ecol.

[CR8] Bio-based Industries (2014) A public-private partnership on bio-based industries combining BBI (H2020) and European structural and investment funds (ESIF) to deploy the European bioeconomy

[CR9] Bjørn A, Hauschild MZ (2013). Absolute versus relative environmental sustainability: what can the cradle-to-cradle and eco-efficiency concepts learn from each other? Bjørn and Hauschild Cradle to Cradle versus Eco-efficiency. J Ind Ecol.

[CR10] Bjørn A, Margni M, Roy PO (2016). A proposal to measure absolute environmental sustainability in life cycle assessment. Ecol Indic.

[CR11] Brockmann D, Gérand Y, Park C (2021). Wastewater treatment using oxygenic photogranule-based process has lower environmental impact than conventional activated sludge process. Bioresour Technol.

[CR12] Brunklaus B, Rex E, Carlsson E, Berlin J (2018). The future of Swedish food waste: an environmental assessment of existing and prospective valorization techniques. J Clean Prod.

[CR13] Bugge MM, Hansen T, Klitkou A (2016) What is the bioeconomy? A review of the literature. Sustain 8. 10.3390/su8070691

[CR14] Chandrakumar C, McLaren SJ (2018). Towards a comprehensive absolute sustainability assessment method for effective Earth system governance: defining key environmental indicators using an enhanced-DPSIR framework. Ecol Indic.

[CR15] Changwichan K, Silalertruksa T, Gheewala SH (2018) Eco-efficiency assessment of bioplastics production systems and end-of-life options. Sustain 10. 10.3390/su10040952

[CR16] Chen W, Oldfield TL, Cinelli P (2020). Hybrid life cycle assessment of potato pulp valorisation in biocomposite production. J Clean Prod.

[CR17] Collins A, Galli A, Patrizi N, Pulselli FM (2018). Learning and teaching sustainability: the contribution of ecological footprint calculators. J Clean Prod.

[CR18] Cooper DR, Gutowski TG (2018). Prospective environmental analyses of emerging technology: a critique, a proposed methodology, and a case study on incremental sheet forming. J Ind Ecol.

[CR19] Cortés A, Moreira MT, Feijoo G (2019). Integrated evaluation of wine lees valorization to produce value-added products. Waste Manag.

[CR20] Cucurachi S, Van Der Giesen C, Guinée J (2018) Ex-ante LCA of emerging technologies. In: Procedia CIRP. Elsevier B.V., pp 463–468

[CR21] D’Amato D, Bartkowski B, Droste N (2020). Reviewing the interface of bioeconomy and ecosystem service research. Ambio.

[CR22] David G, Croxatto Vega G, Sohn J (2020). Using life cycle assessment to quantify the environmental benefit of upcycling vine shoots as fillers in biocomposite packaging materials. Int J Life Cycle Assess.

[CR23] Dente SMR, Kayo C, Aoki-Suzuki C et al (2020) Life cycle environmental impact assessment of biomass materials in Japan. J Clean Prod 257. 10.1016/j.jclepro.2020.120388

[CR24] Dietz T, Börner J, Förster JJ, von Braun J (2018) Governance of the bioeconomy: a global comparative study of national bioeconomy strategies. Sustain 10. 10.3390/su10093190

[CR25] Duan N, Khoshnevisan B, Lin C et al (2020) Life cycle assessment of anaerobic digestion of pig manure coupled with different digestate treatment technologies. Environ Int 137. 10.1016/j.envint.2020.105522 10.1016/j.envint.2020.105522 32007689

[CR26] Durão V, Silvestre JD, Mateus R, de Brito J (2020). Assessment and communication of the environmental performance of construction products in Europe: comparison between PEF and EN 15804 compliant EPD schemes. Resour Conserv Recycl.

[CR27] Durkin A, Taptygin I, Kong Q (2019). Scale-up and sustainability evaluation of biopolymer production from citrus waste offering carbon capture and utilisation pathway. ChemistryOpen.

[CR28] EC-JRC (2010) JRC: Annual Report 2010

[CR29] Ekman A, Campos M, Lindahl S (2013). Bioresource utilisation by sustainable technologies in new value-added biorefinery concepts - two case studies from food and forest industry. J Clean Prod.

[CR30] El-Chichakli B (2016) Five cornerstones of a global bioeconomy 10.1038/535221a 27411618

[CR31] Esnouf A, Heijungs R, Coste G (2019). A tool to guide the selection of impact categories for LCA studies by using the representativeness index. Sci Total Environ.

[CR32] European Commission (2018) A sustainable Bioeconomy for Europe: strengthening the connection between economy, society and the environment

[CR33] European Commission (2014) Towards a circular economy: a zero waste programme for Europe

[CR34] Farzad S, Mandegari MA, Guo M et al (2017) Multi-product biorefineries from lignocelluloses: a pathway to revitalisation of the sugar industry? Biotechnol Biofuels 10. 10.1186/s13068-017-0761-9 10.1186/s13068-017-0761-9 PMC5387292 28400858

[CR35] Finnegan W, Yan M, Holden NM, Goggins J (2017) LCA for agricultural practices and biobased industrial products a review of environmental life cycle assessment studies examining cheese production. Int J Life Cycle Assess 1773–1787

[CR36] Forte A, Dourado F, Mota A (2021). Life cycle assessment of bacterial cellulose production. Int J Life Cycle Assess.

[CR37] Forte A, Zucaro A, Basosi R, Fierro A (2016) LCA of 1,4-butanediol produced via direct fermentation of sugars from wheat straw feedstock within a territorial biorefinery. Materials (Basel) 9. 10.3390/MA9070563 10.3390/ma9070563 PMC5456926 28773687

[CR38] Fridrihsone A, Romagnoli F, Kirsanovs V, Cabulis U (2020) Life cycle assessment of vegetable oil based polyols for polyurethane production. J Clean Prod 266. 10.1016/j.jclepro.2020.121403

[CR39] Gaber K, Rösch C (2021). Biondi N (2021) Life cycle assessment of total fatty acid (TFA) production from microalgae nannochloropsis oceanica at different sites and under different sustainability scenarios. BioEnergy Res.

[CR40] Gadkari S, Kumar D, Qin Z, hao,  (2021). Life cycle analysis of fermentative production of succinic acid from bread waste. Waste Manag.

[CR41] Gallo Stampino P, Riva L, Punta C (2021). Comparative life cycle assessment of cellulose nanofibres production routes from virgin and recycled raw materials. Molecules.

[CR42] Gonzalez-Garcia S, Gullón B, Moreira MT (2018). Environmental assessment of biorefinery processes for the valorization of lignocellulosic wastes into oligosaccharides. J Clean Prod.

[CR43] Gottinger A, Ladu L, Quitzow R (2020). Studying the transition towards a circular bioeconomy—a systematic literature review on transition studies and existing barriers. Sustain.

[CR44] Greenpeace International GWECS (2015) 100% renewable energy for all

[CR45] Guin J (2001). Announcing a new LCA guide editorial : announcing a n e w LCA guide handbook on life cycle assessment - operational guide to the ISO standards. Int J.

[CR46] Gullón P, Gullón B, Dávila I (2018). Comparative environmental life cycle assessment of integral revalorization of vine shoots from a biorefinery perspective. Sci Total Environ.

[CR47] Hakovirta M, Denuwara N, Bharathi S (2020). The importance of diversity on boards of directors’ effectiveness and its impact on innovativeness in the bioeconomy. Humanit Soc Sci Commun.

[CR48] Hanserud OS, Cherubini F, Øgaard AF (2018). Choice of mineral fertilizer substitution principle strongly influences LCA environmental benefits of nutrient cycling in the agri-food system. Sci Total Environ.

[CR49] Hildebrandt J (2018) Revealing the environmental advantages of industrial symbiosis in wood-based bioeconomy networks an assessment from a life cycle perspective. J Ind Ecol 23. 10.1111/jiec.12818

[CR50] Hildebrandt J, Budzinski M, Nitzsche R (2019). Assessing the technical and environmental performance of wood-based fiber laminates with lignin based phenolic resin systems Jakob. Resour Conserv Recycl.

[CR51] Huang X, Shi B, Wang S (2021). Mitigating environmental impacts of milk production via integrated maize silage planting and dairy cow breeding system: a case study in China. J Clean Prod.

[CR52] ILCD (2010) ILCD handbook - European platform on life cycle

[CR53] International Council of Chemical Associations (2019) How to know if and when it ’ s time to commission a life cycle assessment

[CR54] ISO 14044 (2006) Environmental management — life cycle assessment — requirements and guidelines ISO14044. Br Stanards 3

[CR55] Ita-Nagy D, Vázquez-Rowe I, Kahhat R (2020). Life cycle assessment of bagasse fiber reinforced biocomposites. Sci Total Environ.

[CR56] Järviö N, Maljanen NL, Kobayashi Y (2021). An attributional life cycle assessment of microbial protein production: a case study on using hydrogen-oxidizing bacteria. Sci Total Environ.

[CR57] Joglekar SN, Pathak PD, Mandavgane SA, Kulkarni BD (2019). Process of fruit peel waste biorefinery: a case study of citrus waste biorefinery, its environmental impacts and recommendations. Environ Sci Pollut Res.

[CR58] Karp A, Beale MH, Beaudoin F et al (2015) Growing innovations for the bioeconomy. Nat. Plants 1 10.1038/nplants.2015.193 27251725

[CR59] Khoshnevisan B, Tabatabaei M, Tsapekos P et al (2020) Environmental life cycle assessment of different biorefinery platforms valorizing municipal solid waste to bioenergy, microbial protein, lactic and succinic acid. Renew Sustain Energy Rev 117. 10.1016/j.rser.2019.109493

[CR60] Koesling M, Kvadsheim NP, Halfdanarson J (2021). Environmental impacts of protein-production from farmed seaweed: comparison of possible scenarios in Norway. J Clean Prod.

[CR61] Kopsahelis A, Kourmentza C., Zafiri C KM (2018) Gate-to-gate life cycle assessment of biosurfactants and bioplasticizers production via biotechnological exploitation of fats and waste oils

[CR62] Krzyżaniak M, Stolarski MJ, Warmiński K (2020) Life cycle assessment of giant Miscanthus: production on marginal soil with various fertilisation treatments. Energies 13. 10.3390/en13081931

[CR63] Lam CM, Yu IKM, Hsu SC, Tsang DCW (2018). Life-cycle assessment on food waste valorisation to value-added products. J Clean Prod.

[CR64] Lehmann LM, Borzęcka M, Żyłowska K et al (2020) Environmental impact assessments of integrated food and non-food production systems in Italy and Denmark. Energies 13.10.3390/en13040849

[CR65] Leoussis J, Brzezicka P (2017) Access-to-finance conditions for investments in bio-based industries and the Blue Economy. DG Res Innov Eur Com 126

[CR66] Lettner M, Solt P, Rößiger B et al (2018) From wood to resin-identifying sustainability levers through hotspotting lignin valorisation pathways. Sustain 10. 10.3390/su10082745

[CR67] Maga D, Thonemann N, Hiebel M et al (2019) Comparative life cycle assessment of first- and second-generation ethanol from sugarcane in Brazil. LCA Energy Syst Food Prod 266–280

[CR68] Manandhar A, Shah A (2017). Life cycle assessment of feedstock supply systems for cellulosic biorefineries using corn stover transported in conventional bale and densified pellet formats. J Clean Prod.

[CR69] Mandegari MA, Farzad S, van Rensburg E, Görgens JF (2017). Multi-criteria analysis of a biorefinery for co-production of lactic acid and ethanol from sugarcane lignocellulose. Biofuels, Bioprod Biorefining.

[CR70] Martínez-Blanco J, Inaba A, Finkbeiner M (2015). Scoping organizational LCA—challenges and solutions. Int J Life Cycle Assess.

[CR71] Matthews SH, Hendrickson CT, Matthews DH (2014) Life cycle assessment: quantitative approaches for decisions that matter

[CR72] McCormick K, Kautto N (2013). The bioeconomy in europe: an overview. Sustainability.

[CR73] McManus MC, Taylor CM (2015). The changing nature of life cycle assessment. Biomass Bioenerg.

[CR74] Meyer R (2017) Bioeconomy strategies: contexts, visions, guiding implementation principles and resulting debates. Sustain 9. 10.3390/su906103

[CR75] Ministry of Infrastructure and the Environment (2011) Usability of life cycle assessment for cradle to cradle purposes position paper. Focus on sustainability, innovation and international Usability of LCA for C2C purposes.

[CR76] Mohammadi A, Cowie AL, Anh Mai TL (2017). Climate-change and health effects of using rice husk for biochar-compost: comparing three pyrolysis systems. J Clean Prod.

[CR77] Mongili B, Abdel Azim A, Fraterrigo Garofalo S (2021). Novel insights in dimethyl carbonate-based extraction of polyhydroxybutyrate (PHB). Biotechnol Biofuels.

[CR78] Morão A, de Bie F (2019). Life cycle impact assessment of polylactic acid (PLA) produced from sugarcane in Thailand. J Polym Environ.

[CR79] Moretti C, Hamelin L, Jakobsen LG (2021). Cradle-to-grave life cycle assessment of single-use cups made from PLA. PP and PET Resour Conserv Recycl.

[CR80] Moretti C, Junginger M, Shen L (2020). Environmental life cycle assessment of polypropylene made from used cooking oil. Resour Conserv Recycl.

[CR81] Muench S, Guenther E (2013). A Systematic Review of Bioenergy Life Cycle Assessments.

[CR82] Myllyviita T, Sironen S, Saikku L et al (2019) Assessing biodiversity impacts in life cycle assessment framework - comparing approaches based on species richness and ecosystem indicators in the case of Finnish boreal forests. J Clean Prod 236. 10.1016/j.jclepro.2019.117641

[CR83] Ncube A, Fiorentino G, Colella M, Ulgiati S (2021). Upgrading wineries to biorefineries within a circular economy perspective: an Italian case study. Sci Total Environ.

[CR84] Negro V, Ruggeri B, Fino D, Tonini D (2017). Life cycle assessment of orange peel waste management. Resour Conserv Recycl.

[CR85] Nitkiewicz T, Wojnarowska M, Sołtysik M et al (2020) How sustainable are biopolymers? Findings from a life cycle assessment of polyhydroxyalkanoate production from rapeseed-oil derivatives. Sci Total Environ 749. 10.1016/j.scitotenv.2020.141279 10.1016/j.scitotenv.2020.141279 32818854

[CR86] Ntimugura F, Vinai R, Harper AB, Walker P (2021). Environmental performance of miscanthus-lime lightweight concrete using life cycle assessment: application in external wall assemblies A NOTE ON VERSIONS Environmental performance of miscanthus-lime lightweight concrete using life cycle assessment: Applic. Sustain Mater Technol.

[CR87] Ögmundarson Ó, Sukumara S, Laurent A, Fantke P (2020). Environmental hotspots of lactic acid production systems. GCB Bioenergy.

[CR88] Oldfield TL, White E, Holden NM (2018). The implications of stakeholder perspective for LCA of wasted food and green waste. J Clean Prod.

[CR89] Oldfield TL, White E, Holden NM (2016). An environmental analysis of options for utilising wasted food and food residue. J Environ Manage.

[CR90] Oliveira M, Cocozza A, Zucaro A (2021). Circular economy in the agro-industry: integrated environmental assessment of dairy products. Renew Sustain Energy Rev.

[CR91] Parajuli R, Knudsen MT, Birkved M (2017). Environmental impacts of producing bioethanol and biobased lactic acid from standalone and integrated biorefineries using a consequential and an attributional life cycle assessment approach. Sci Total Environ.

[CR92] Parsons S, Allen MJ, Abeln F (2019). Sustainability and life cycle assessment (LCA) of macroalgae-derived single cell oils. J Clean Prod.

[CR93] Pehme S, Veromann E, Hamelin L (2017). Environmental performance of manure co-digestion with natural and cultivated grass – a consequential life cycle assessment. J Clean Prod.

[CR94] Pergola M, Gialdini A, Celano G (2018). An environmental and economic analysis of the wood-pellet chain: two case studies in Southern Italy. Int J Life Cycle Assess.

[CR95] Porcelli R, Dotto F, Pezzolesi L et al (2020) Comparative life cycle assessment of microalgae cultivation for non-energy purposes using different carbon dioxide sources. Sci Total Environ 721. 10.1016/j.scitotenv.2020.137714 10.1016/j.scitotenv.2020.137714 32171140

[CR96] Posen ID, Jaramillo P, Griffin WM (2016). Uncertainty in the life cycle greenhouse gas emissions from U.S. production of three biobased polymer families. Environ Sci Technol.

[CR97] Pryshlakivsky J, Searcy C (2013). Fifteen years of ISO 14040: a review. J Clean Prod.

[CR98] Pyka A, Cardellini G, van Meijl H, Verkerk PJ (2022). Modelling the bioeconomy: emerging approaches to address policy needs. J Clean Prod.

[CR99] Rehl T, Lansche J, Müller J (2012). Life cycle assessment of energy generation from biogas - attributional vs. consequential approach. Renew Sustain Energy Rev.

[CR100] Righi S, Baioli F, Samorì C (2017). A life cycle assessment of poly-hydroxybutyrate extraction from microbial biomass using dimethyl carbonate. J Clean Prod.

[CR101] Risse M, Weber-Blaschke G, Richter K (2019). Eco-efficiency analysis of recycling recovered solid wood from construction into laminated timber products. Sci Total Environ.

[CR102] Rønning A, Brekke A (2013) Life cycle assessment (LCA) of the building sector: strengths and weaknesses. In: Eco-Efficient Construction and Building Materials: Life Cycle Assessment (LCA), Eco-Labelling and Case Studies. Elsevier Inc., pp 63–83

[CR103] Rønning A, Brekke A (2014) Life cycle assessment (LCA) of the building sector: strengths and weaknesses. In: Eco-Efficient Construction and Building Materials: Life Cycle Assessment (LCA), Eco-Labelling and Case Studies. Elsevier Inc., pp 63–83

[CR104] Roy P, Defersha F, Rodriguez-Uribe A (2020). Evaluation of the life cycle of an automotive component produced from biocomposite. J Clean Prod.

[CR105] Sadhukhan J, Martinez-Hernandez E (2017). Material flow and sustainability analyses of biorefining of municipal solid waste. Bioresour Technol.

[CR106] Salim I, González-García S, Feijoo G, Moreira MT (2019). Assessing the environmental sustainability of glucose from wheat as a fermentation feedstock. J Environ Manage.

[CR107] Sandén BA, Karlström M (2007). Positive and negative feedback in consequential life-cycle assessment. J Clean Prod.

[CR108] Santagata R, Ripa M, Genovese A, Ulgiati S (2021) Food waste recovery pathways: challenges and opportunities for an emerging bio-based circular economy. A systematic review and an assessment. J Clean Prod 286:125490. 10.1016/j.jclepro.2020.125490

[CR109] Schmidt Rivera XC, Gallego-Schmid A, Najdanovic-Visak V, Azapagic A (2020) Life cycle environmental sustainability of valorisation routes for spent coffee grounds: from waste to resources. Resour Conserv Recycl 157. 10.1016/j.resconrec.2020.104751

[CR110] Seghetta M, Romeo D, D’Este M (2017). Seaweed as innovative feedstock for energy and feed – evaluating the impacts through a Life Cycle Assessment. J Clean Prod.

[CR111] Seghetta M, Tørring D, Bruhn A, Thomsen M (2016). Bioextraction potential of seaweed in Denmark - an instrument for circular nutrient management. Sci Total Environ.

[CR112] Severo IA, Deprá MC, Dias RR et al (2020) Bio-combustion of petroleum coke: the process integration with photobioreactors. Part II – Sustainability metrics and bioeconomy. Chem Eng Sci 213. 10.1016/j.ces.2019.115412

[CR113] Shemfe M, Gadkari S, Yu E (2018). Life cycle, techno-economic and dynamic simulation assessment of bioelectrochemical systems: a case of formic acid synthesis. Bioresour Technol.

[CR114] Silalertruksa T, Gheewala SH (2019) Competitive use of sugarcane for food, fuel, and biochemical through the environmental and economic factors

[CR115] Soimakallio S, Saikku L, Valsta L, Pingoud K (2016). Climate change mitigation challenge for wood utilization-the case of Finland. Environ Sci Technol.

[CR116] Somers MD, Quinn JC (2019) Sustainability of carbon delivery to an algal biorefinery : a techno-economic and life-cycle assessment. J CO2 Util 30:193–204. 10.1016/j.jcou.2019.01.007

[CR117] Sommerhuber PF, Wenker JL, Rüter S, Krause A (2017). Life cycle assessment of wood-plastic composites: analysing alternative materials and identifying an environmental sound end-of-life option. Resour Conserv Recycl.

[CR118] Stegmann P, Londo M, Junginger M (2020) The circular bioeconomy: its elements and role in European bioeconomy clusters. Resour Conserv Recycl X 6:100029. 10.1016/J.RCRX.2019.100029

[CR119] Streeck J, Hank C, Neuner M (2018). Bio-electrochemical conversion of industrial wastewater-COD combined with downstream methanol synthesis-an economic and life cycle assessment. Green Chem.

[CR120] Suhariyanto TT, Wahab DA, Rahman MNA (2017). Multi-life cycle assessment for sustainable products: a systematic review. J Clean Prod.

[CR121] Ryding S-O (1997). Environmental management - life cycle assessment - life cycle impact assessment. Editor ISO.

[CR122] The International Advisory Council on Global Bioeconomy (2020) Global bioeconomy policy report (IV): a decade of bioeconomy policy development around the world

[CR123] Thielemann AK, Smetana S, Pleissner D (2021). Life cycle assessment of hetero- and phototrophic as well as combined cultivations of Galdieria sulphuraria. Bioresour Technol.

[CR124] Thonemann N, Schulte A (2019). From laboratory to industrial scale: a prospective LCA for electrochemical reduction of CO2 to formic acid. Environ Sci Technol.

[CR125] Transport & Environment, BirdLife International (2016) How much sustainable biomass does Europe have in 2030? Report 1–7

[CR126] Ubando AT, Felix CB, Chen WH (2020) Biorefineries in circular bioeconomy: a comprehensive review. Bioresour Technol 299. 10.1016/j.biortech.2019.122585 10.1016/j.biortech.2019.122585 31901305

[CR127] Urmetzer S, Lask J, Vargas-Carpintero R, Pyka A (2020). Learning to change: transformative knowledge for building a sustainable bioeconomy. Ecol Econ.

[CR128] van Oirschot R, Thomas JBE, Gröndahl F (2017). Explorative environmental life cycle assessment for system design of seaweed cultivation and drying. Algal Res.

[CR129] Vega GC, Sohn J, Bruun S et al (2019) Maximizing environmental impact savings potential through innovative biorefinery alternatives: an application of the TM-LCA framework for regional scale impact assessment. Sustain 11.10.3390/su11143836

[CR130] Wagner M, Kiesel A, Hastings A et al (2017) Novel miscanthus germplasm-based value chains: a life cycle assessment. Front Plant Sci 8. 10.3389/fpls.2017.00990 10.3389/fpls.2017.00990 PMC5462955 28642784

[CR131] Weidema BP, Ekvall T, Heijungs R (2009) Guidelines for application of deepened and broadened LCA

[CR132] Wohlfahrt J, Ferchaud F, Gabrielle B (2019). Characteristics of bioeconomy systems and sustainability issues at the territorial scale. A Review J Clean Prod.

[CR133] Yan MJ, Humphreys J, Holden NM (2011). An evaluation of life cycle assessment of European milk production. J Environ Manage.

[CR134] Zhang D, del Rio-Chanona EA, Shah N (2018). Life cycle assessments for biomass derived sustainable biopolymer & energy co-generation. Sustain Prod Consum.

[CR135] Zhang D, del Rio-Chanona EA, Wagner JL, Shah N (2018). Life cycle assessments of bio-based sustainable polylimonene carbonate production processes. Sustain Prod Consum.

[CR136] Zhang X, Border A, Goosen N, Thomsen M (2021). Environmental life cycle assessment of cascade valorisation strategies of South African macroalga Ecklonia maxima using green extraction technologies. Algal Res.

[CR137] Zhang Y, Hu G, Brown RC (2014). Life cycle assessment of commodity chemical production from forest residue via fast pyrolysis. Int J Life Cycle Assess.

[CR138] Zhang YI, Singh S, Bakshi BR (2010). Accounting for ecosystem services in life cycle assessment part I: a critical review. Environ Sci Technol.

[CR139] Zumsteg JM, Cooper JS, Noon MS (2012) Systematic review checklist: a standardized technique for assessing and reporting reviews of life cycle assessment data. J Ind Ecol 16. 10.1111/j.1530-9290.2012.00476 10.1111/j.1530-9290.2012.00476.x PMC4461004 26069437

